# An unexpected genetic diversity pattern and a complex demographic history of a rare medicinal herb, Chinese asparagus (*Asparagus cochinchinensis*) in Korea

**DOI:** 10.1038/s41598-019-46275-9

**Published:** 2019-07-05

**Authors:** Soo-Rang Lee, Han-Sol Park, Bo-Yun Kim, Jung-Hoon Lee, Qiang Fan, John F. Gaskin, Young-Dong Kim

**Affiliations:** 1 Multidisciplinary Genome Institute, Hallym University, Chuncheon, 24252 South Korea; 2 Department of Life Sciences, Hallym University, Chuncheon, 24252 South Korea; 3 National Institute of Biological Resources, Incheon, 22689 South Korea; 4 State Key Laboratory of Biocontrol and Guangdong Provincial Key Laboratory of Plant Resources, Sun Yat-sen University, Guangzhou, 510275 China; 5 USDA-ARS, 1500 North Central Avenue, Sidney, Montana 59270 USA

**Keywords:** Molecular evolution, Genetic variation, Genotype, Plant evolution

## Abstract

Range-wide population studies of wide spread species are often associated with complex diversity patterns resulting from genetically divergent evolutionary significant units (ESUs). The compound evolutionary history creating such a pattern of diversity can be inferred through molecular analyses. *Asparagus cochinchinensis*, a medicinally important perennial herb, is in decline due to overharvesting in Korea. Eight *A*. *cochinchinensis* populations in Korea and three populations from neighboring countries (China, Japan and Taiwan) were examined using nine nuclear microsatellite loci and three chloroplast microsatellite loci to characterize molecular diversity patterns. The average within-population diversity was limited likely due to long-term bottlenecks observed in all eight populations. High pairwise F_ST_ values indicated that the populations have largely diverged, but the divergences were not correlated with geographic distances. Clustering analyses revealed a highly complex spatial structure pattern associated with two ESUs. Approximate Bayesian Computation (ABC) suggests that the two ESUs split about 21,000 BP were independently introduced to Korea approximately 1,800 years ago, and admixed in secondary contact zones. The two ESUs found in our study may have different habitat preferences and growth conditions, implying that the two genetically divergent groups should be considered not only for conservation and management but also for breeding programs in agricultural areas.

## Introduction

Population-level genetic studies for species across wide ranges often reveal that the species consist of multiple Evolutionary Significant Units (ESUs) or even harbor a cryptic species^1^. The past and recent species range expansions with accompanied demographic changes may result in extreme patterns of population divergence^2,3^. For example, quaternary glacial and interglacial oscillations have had major impacts on the evolution of divergent lineages as ice sheets isolated populations in distant refugia^4^. Allopatric divergence might have further led to establishment of ESUs or cryptic species from a range of glacial refugia^4,5^. Populations that experience such dramatic evolutionary events often exhibit complex patterns of genetic diversity. However, caution is warranted because similar genetic patterns can also be drawn from gene flow, selection and random effects in combination with life-history characteristics such as reproduction and dispersal modes^6^.

One of the most commercially important genera, *Asparagus* L. (monocot; Asparagaceae), consists of ~120 species including edible crops: *A*. *officinalis* & *A*. *albus*, and ornamental and/or medicinal herbs: *A*. *asparagoides*, *A*. *falcatus*, *A*. *setaceus*, *A*. *scandens*^7–9^. *Asparagus* is an Old World genus with diverse life forms (herbs, shrubs and climbers). It is distributed across the Mediterranean, semi-arid and arid environments^9^. Notably, some species in the genus are endangered, whereas several others pose great threats to biodiversity as invasive weeds^8,10^. However, the basic biology as well as the phylogenetic relationships of most species in the genus have remained uncertain, except for the few well-known species listed above^8,9,11^. The limited knowledge on most species in the genus raises great challenges in at least two important aspects: (1) finding proper target species to improve the important crop, garden asparagus (*A*. *officinalis*)^11,12^ and (2) management of notorious invaders (e.g., *A*. *aethiopicus*, *A*. *africanus*, and *A*. *virgatus*) causing biodiversity losses^10^. Empirical studies of ecological characteristics and evolutionary relationships will provide valuable insights for biodiversity management and enhancement of crop production.

*Asparagus cochinchinensis* (Lour.) Merr. is a perennial herb that is widely distributed from temperate (China, Korea, Japan) to tropical Asia (Laos and Vietnam)^13^. This species is one of the closest relatives to the commercially important crop, garden asparagus. The tuberous roots of the plants have been widely used as medicine in many oriental countries^11,12,14–16^. Although global population trend information is completely lacking for the species^16^, Korean populations are thought to be in decline due to over-harvesting^14^. In China, *A*. *cochinchinensis* widely occur in thinly forested slopes, roadsides and waste fields^13^, whereas the species is mainly found near seashores in Korea and Japan^14,17^. A recent study revealed that the plants mostly occur along the west coast but not along the east coast in Korea^14^. The large difference in habitat characteristics and distribution patterns between China and Korea/Japan suggests the possibility of multiple ESUs accompanied by a series of demographic changes for *A*. *cochinchinensis* in Korea^14,18^.

By understanding the distribution of genetic diversity, history of demographic changes, i.e., the introductions, genetic bottlenecks and admixtures can be inferred. An advanced model-based approach, Approximate Bayesian Computation (ABC), offers a powerful tool to investigate genetic data. ABC method is equipped to compare multiple evolutionary hypotheses and estimate the parameters of hypothetical scenarios with uncertainty incorporated^19,20^. Coupled with the traditional population genetics approach, ABC method provides more sophisticated reconstructions of the evolutionary history for a target species.

Here, we employ population genetics tools as well as ABC methods to unravel the complex evolutionary history of *Asparagus cochinchinensis* in Korea. Our specific aims are to (1) investigate the overall spatial genetic diversity pattern, (2) identify ESUs and/or cryptic species, and (3) determine the most probable evolutionary scenario that characterized the current diversity pattern of *A*. *cochinchinensis* populations in Korea. To address these goals properly, we examined genetic diversity patterns over a large geographic scale, including most Korean populations and samples from neighboring countries that might have influence the genetic diversity patterns. The ABC paradigm was applied to test multiple likely hypotheses of the evolutionary history. Population divergences are expected to be low considering the reproductive mode and dispersal capability of the species. However, populations might have been much more differentiated than expected if there are multiple ESUs present. Given the wide range of geographic distribution and the breadth of the habitats, we hypothesize that the distribution of genetic variation will exhibit rather complex patterns, and the complexity will be much higher with the presence of multiple ESUs associated with cryptic diversity. Alternatively, the genetic affinities among populations might reflect environmental and/or geographical differences. However, the spatial and environmental patterns of genetic diversity might not be prominent if there was a series of historical demographic events.

## Results

Genetically identical clones were very rare in all eight populations (150 genets in 158 samples). We found no scoring error in nuclear microsatellite profiles during the genotyping procedure. Null alleles were present for two loci with low (0.1) to moderate (0.35) frequencies. One of the two loci (AC069; see Kim *et al*. 2017 for detailed information about the marker loci)^21^ appeared to have null alleles present in all eight populations while null alleles were present in only two populations for the other locus (AC079). A significant departure from Hardy-Weinberg Equilibrium (HWE) was shown in locus 3 (AC014, high heterozygosity) and locus 8 (AC069; an excess of homozygotes with null allele present). We found that two loci (AC008, AC017) were linked with the remaining 11 loci in most populations (significant linkage disequilibrium, LD, at P < 0.005). Because some genetic analyses such as STRUCTURE and DIYABC depend on strong assumptions of marker independence and HWE, we purged four markers (AC008, AC014, AC017, and AC069) that violated the assumptions from all downstream analyses.

The expected heterozygosity (He), observed heterozygosity (Ho) and number of alleles per population (Na) averaged over individuals varied among the populations (Table 1). He ranged from 0.22 to 0.39 and Na was between 1.44 and 2.56 (Table 1). In most populations, the F_IS_ values were close to zero except for HAM (F_IS_ = 0.34 ± 0.15; the latter number is the Standard Error, SE) and OKN (F_IS_ = 0.31 ± 0.20). We do not present F_IS_ values here because most values were not significantly different from zero. Pairwise F_ST_ values were all statistically significant and differed greatly among population pairs; the lowest was 0.097 for the YGW/TPE pair (see Table 1 for population abbreviations) and the highest was 0.631 for HAM/OKN (Table 2). According to a Mantel test, there was no correlation between genetic divergence (F_ST_) and geographic distance (Euclidean distance; r = 0.01, P = 0.43; Fig. S1).

**Table 1:** Information of locations, sample sizes and summary statistics of genetic diversity for eight *Asparagus cochinchinensis* populations.

Location	Abbreviation	Cluster	N	Lon	Lat	cpSSR		nSSR		
						Nh	Hehap	He[±sd]	Ho[±sd]	Na[±sd]
Yeonggwang, Jeonnam, S. Korea	YGW	Pop5	20	126.409	35.391	3	0.06 [0.06]	0.26 [0.15]	0.22 [0.15]	2.22 [0.44]
Haman, Gyeongnam, S. Korea	HAM	Pop2	20	128.450	35.264	1	0	0.33 [0.17]	0.26 [0.19]	1.44 [0.53]
Wando, Jeonnam, S. Korea	WND	Pop4	18	126.994	34.409	3	0.07 [0.03]	0.35 [0.21]	0.26 [0.17]	1.89 [0.78]
Boryeong, Chungnam, S. Korea	BOR	Pop2	20	126.533	36.283	1	0.03 [0.03]	0.34 [0.17]	0.31 [0.16]	1.67 [0.71]
Namhae, Gyeongnam, S. Korea	NAH	Pop5	20	127.867	34.733	4	0.17 [0.10]	0.32 [0.16]	0.33 [0.22]	2.00 [1.23]
Keelung, Taipei, Taiwan	TPE	Pop6	20	121.920	25.123	7	0.36 [0.17]	0.27 [0.19]	0.24 [0.22]	2.22 [0.83]
Okinawa, Japan	OKN	Pop3	20	127.850	26.500	3	0.15 [0.15]	0.31 [0.29]	0.27 [0.31]	1.56 [1.01]
Guangdong, China	GND	Pop1	20	113.733	25.050	1	0	0.39 [0.24]	0.39 [0.18]	2.56 [1.51]

Cluster-a cluster to which the population is assigned for the DIYABC analysis. N-Sample size. Lat and Lon-geographic coordinates. Nh-number of haplotypes found in each population. Hehap-mean haplotype diversity across three cpSSRs. He and Ho-mean expected and observed heterozygosity over nine nrSSRs. Na-mean number of alleles over nine nrSSRs. sd-standard deviation.

**Table 2:** Estimated pairwise F_ST_ and Φ_PT_ values from nine nrSSRs and three cpSSRs among eight *A*. *cochinchinensis* populations across South Korea, China, Taiwan and Japan.

	YGW	NAH	TPE	HAM	BOR	GND	WND	OKN
YGW	0.000	0.079	0.250	0.026	0.018	0.911	0.000	0.821
NAH	0.278	0.000	0.134	0.096	0.034	0.774	0.054	0.714
TPE	0.097	0.350	0.000	0.283	0.249	0.396	0.211	0.479
HAM	0.514	0.541	0.571	0.000	0.000	1.000	0.006	0.866
BOR	0.448	0.471	0.487	0.247	0.000	0.952	0.002	0.839
GND	0.411	0.474	0.450	0.309	0.160	0.000	0.900	0.688
WND	0.294	0.368	0.355	0.513	0.420	0.317	0.000	0.806
OKN	0.543	0.543	0.573	0.631	0.612	0.544	0.516	0.000

See Table 1 for abbreviations of population locations and sample sizes. Values above the diagonal are the pairwise Φ_PT_ estimates. All values were significantly different from 0 at the *P* < 0.05 level.

**Figure 1:**
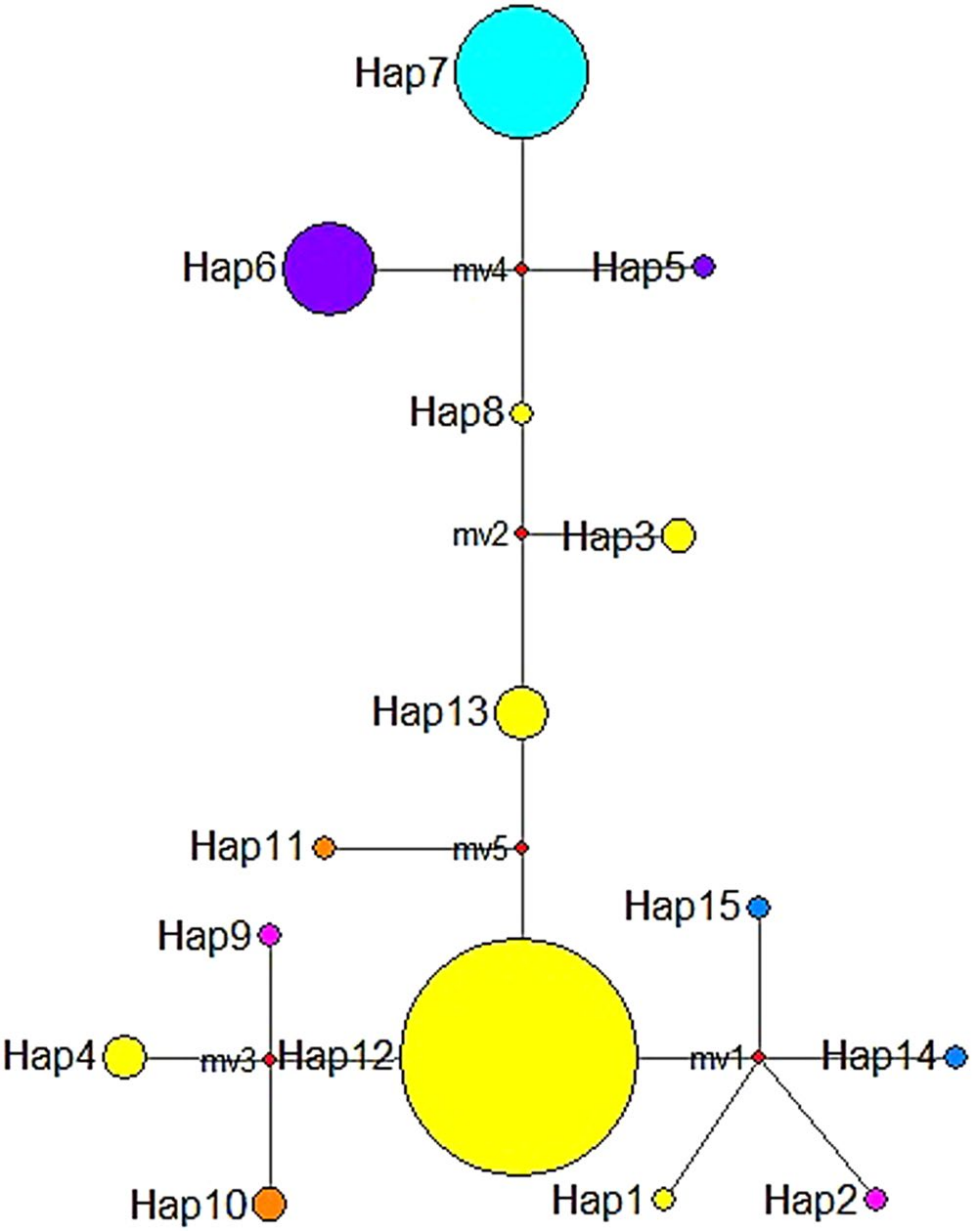
Haplotype network of cpSSR variation for eight *Asparagus cochinchinensis* populations. The median-joining network summarizes the genetic distance among 15 haplotypes, where each circle represents a distinct cpSSR haplotype and the size of the circles is proportional to the frequency of each haplotype (Table S1).

Overall, we found limited haplotype variation (Table 1). With the three cpSSRs, 15 length variations were detected (4–6 alleles per locus), resulting in 15 haplotypes for 158 samples from eight populations (Table S1; Fig. 1). Of the 15 haplotypes, 10 (haplotype codes 1, 2, 3, 5, 8, 9, 10, 11, 14, and 15) were found in a single or in only two individuals (frequency ~1%). About 40% of the low frequency haplotypes were found in population TPE. About 60% of all samples were predominantly assigned to a single haplotype (12), followed by the next most frequent haplotype (7; comprising ~20% of the total sample; Table 1). Haplotype diversity was highest in TPE (see Table 1 for population abbreviations and locations), whereas two populations exhibited haplotype diversity of zero (GND & HAM; Table 1). The pairwise genetic differentiation among the populations varied widely from zero to 1.0 across population pairs (Table 2). The Φ_PT_ values between population pairs within Korea were low (less than 0.1), whereas those estimated for population pairs between Korean populations and the Chinese population (GND) were much higher (0.77–1.00; Table 2). Population differentiation between Korean populations and the Japanese population (OKN) was also very high (0.71–0.87; Table 2). AMOVA results revealed that ~70% of the total haplotype variation stems from regional groups (df = 3; sum squares = 37.21), while less than 30% of the variation comes from within-populations (df = 150; sum squares = 25.19).

**Figure 2:**
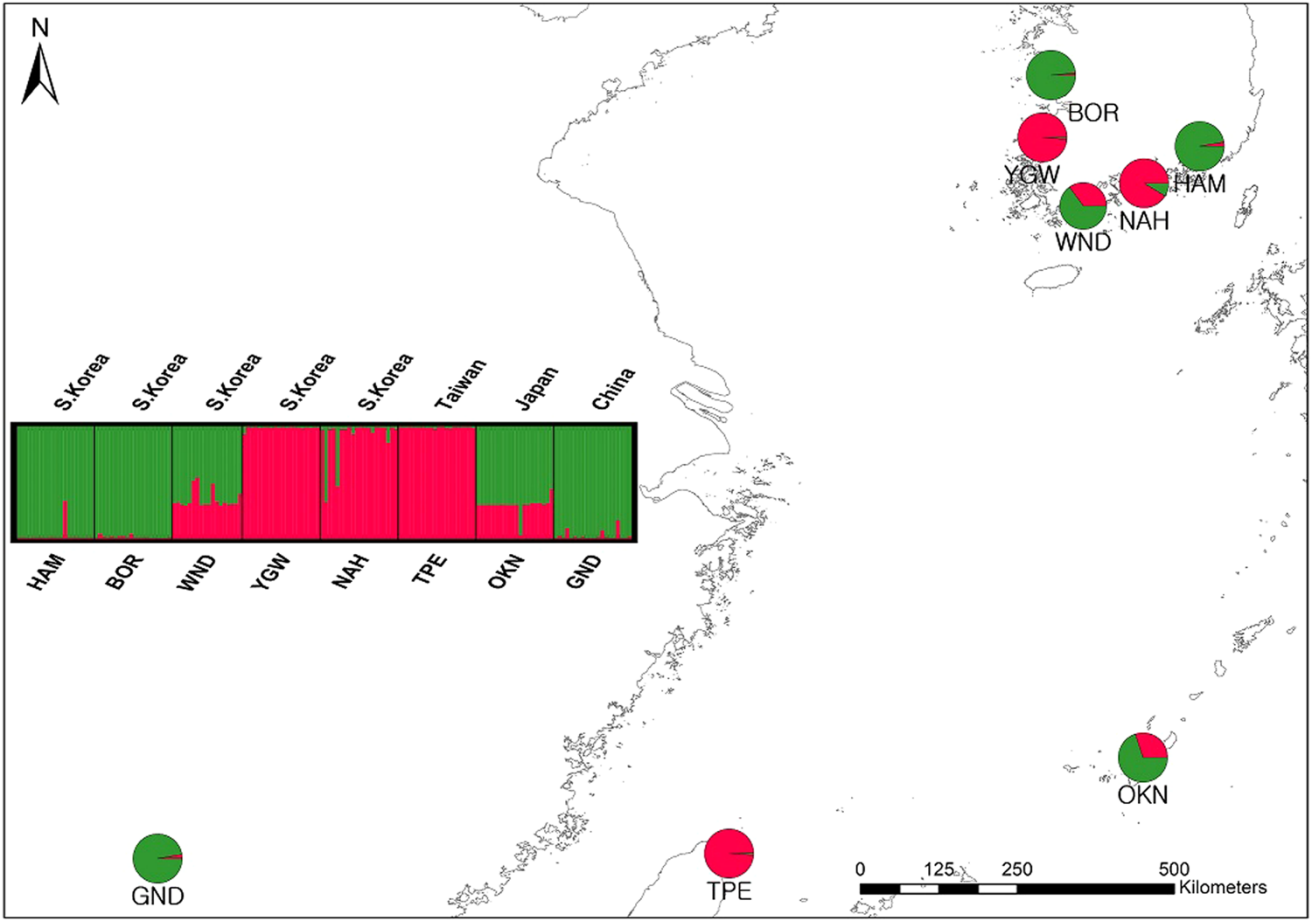
Bayesian model-based clustering analysis of nine microsatellite loci for eight *Asparagus cochinchinensis* populations. The bar plot shows the group assignments of 158 individual genotypes for K = 2 (the optimal number of clusters; Fig. S1). The vertical black lines separate populations. Pie charts on the map depicts the frequency of each cluster in each population. See Table 1 for population abbreviations, sample locations and sample sizes.

K, the number of randomly mating subgroups that best explain our data, was equal to 2 based on delta K as estimated from the STRUCTURE results (Fig. S2). Along with the best K, we present bar plots for K = 2 to 8 to show the clustering patterns with various numbers of K clusters (Fig. S3). The STRUCTURE results for the optimal K (K = 2) clearly show that the populations largely diverge into two groups with an admixture pattern observed in WND and OKN (Fig. 2; see Table 1 for populations details). The Chinese (GND) and Taiwanese (TPE) populations were genetically distinct, while the Japanese population (OKN) shared genetic affinity with both populations (GND & TPE; Fig. 2). Of five Korean populations, two (BOR & HAM) shared alleles with the Chinese population, whereas the other two populations (NAH & YGW) were genetically affiliated with the Taiwanese population (Fig. 2). One southern population, WND, revealed a pattern similar to that of the Japanese population, which is associated with the admixture between the Chinese and Taiwanese populations (Fig. 2). As the K values become larger, YGW and OKN show unique assignment patterns, although overall the split pattern of two groups remains consistent throughout varying K values (Fig. S3).

**Figure 3:**
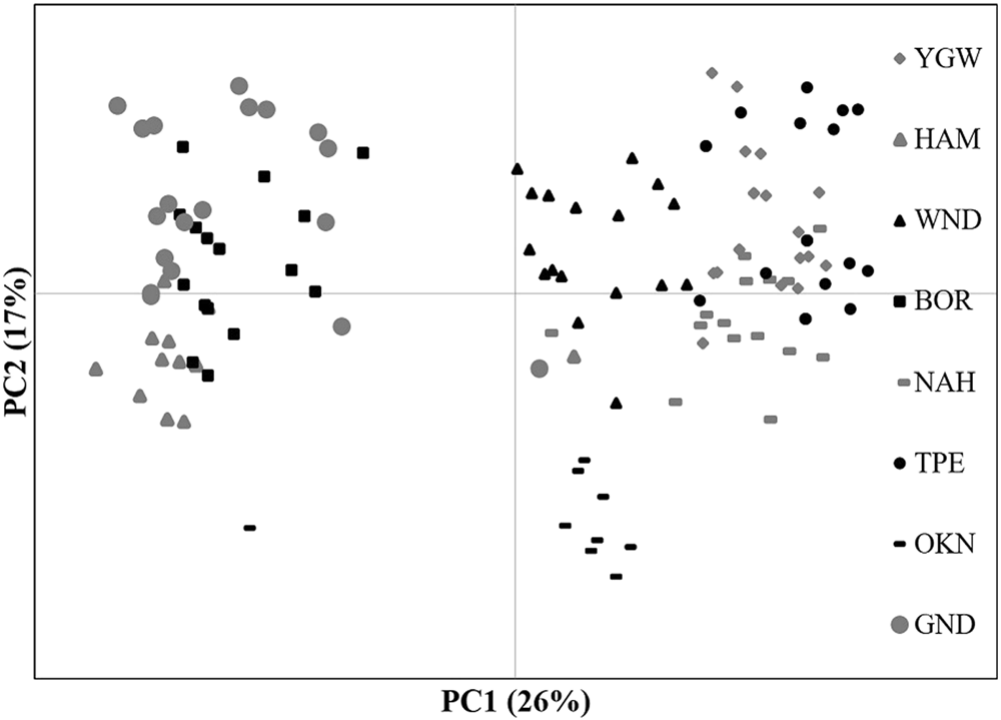
PCoA plot for 158 individuals of *Asparagus cochinchinensis* from eight populations. The first two variance components are plotted. See Table 1 for population abbreviations, sample locations and sample sizes.

PCoA results were consistent with the STRUCTURE results, which largely separated two distinct groups particularly on the first PC axis (PC1, explained 26% of the molecular variance; Fig. 3). Likewise, BOR and HAM were clustered with GND, whereas NAH and YGW were clustered with TPE (Fig. 3). OKN and WND were positioned between the two clusters, although they were rather closer to the latter cluster with TPE (Fig. 3). A *post-hoc* AMOVA analysis with the arranged groups based on both STRUCTURE and PCoA analyses (3 groups: BOR, GND & HAM; NAH, TPE & YGW; OKN & WND) revealed that the populations were genetically more diverged within groups (F_SC_ = 0.25) than across groups (F_CT_ = 0.31; Table 3). The genetic variance was largely partitioned to within individuals (47%), followed by among groups (25%; Table 3).

**Table 3:** Analysis of molecular variance (AMOVA) in *A*. *cochinchinensis* based on nine microsatellite loci.

Source	Sum of squares	Variance components	Percentage of variation	Fixation index
Among groups (F_CT_)	126.504	0.445	24.658	0.247
Among population within groups (F_SC_)	87.069	0.415	22.984	0.305
Among individuals within populations (F_IS_)	155.521	0.093	5.149	0.098
Within individuals (F_IT_)	134.500	0.853	47.209	0.528

Genetic groups identified from STRUCTURE and PCoA results were used for hierarchical partitioning of genetic variance. All variance components were statistically significant (*P* < 0.01).

Based on both Sign and Wilcoxon tests, there was no evidence of recent population bottlenecks for all eight populations under both IAM and SMM mutation models (Table 4). In contrast, we found signatures of long-term bottlenecks in all populations. G-W indices (M) were much lower than the critical value of M = 0.73, as estimated from the data (Table 4). Of the total 28 population pairs (56 combinations of pairs for both directions), approximately 90% of population pairs showed ~1% or fewer migrants per generation (Table S2). We found a slightly higher frequency of migrants between two population pairs (BOR-HAM & NAH-WND; ~5% or less). Overall, the migration rates were approximately equal for both directions, but a few population pairs showed migration rates which were significantly skewed toward one direction (Table S2). Notably, the Chinese, Taiwanese and Korean population pairs, TPE-YGW and GND-BOR, exhibited the highest migration rates (~20%; Table S2). The frequencies of migrants from the Chinese and Taiwanese populations (TPE and GND to Korean YGW and BOR were fairly high (~20%), whereas frequencies of migrants in the opposite directions were much lower (~2% or less; Table S2).

**Table 4:** Summary of tests for recent and past bottlenecks in *A*. *cochinchinensis* populations: G-W index is Garza-Williamson index, known as the M-ratio, the ratio of the number of alleles to the allele size range.

Population	G-W index[±sd]	P (Sign test)		P (Wilcoxon test)		Mode shift
		IAM	SMM	IAM	SMM	
YGW	0.44 [0.18]	0.16	0.08	0.57	0.25	no
HAM	0.53 [0.18]	0.21	0.26	0.19	0.31	no
WND	0.41 [0.16]	0.31	0.60	0.16	1.00	no
BOR	0.37 [0.15]	0.41	0.53	0.16	0.81	no
NAH	0.39 [0.14]	0.42	0.36	1.00	0.69	no
TPE	0.46 [0.18]	0.45	0.13	0.74	0.25	no
OKN	0.52 [0.13]	0.51	0.47	1.00	0.25	no
GND	0.42 [0.20]	0.21	0.56	0.58	0.81	no

The significant P-values of Sign and Wilcoxon signed-rank tests are from the tests for excess or deficit of heterozygosity across nine microsatellite loci under the IAM and SMM mutation models.

**Figure 4:**
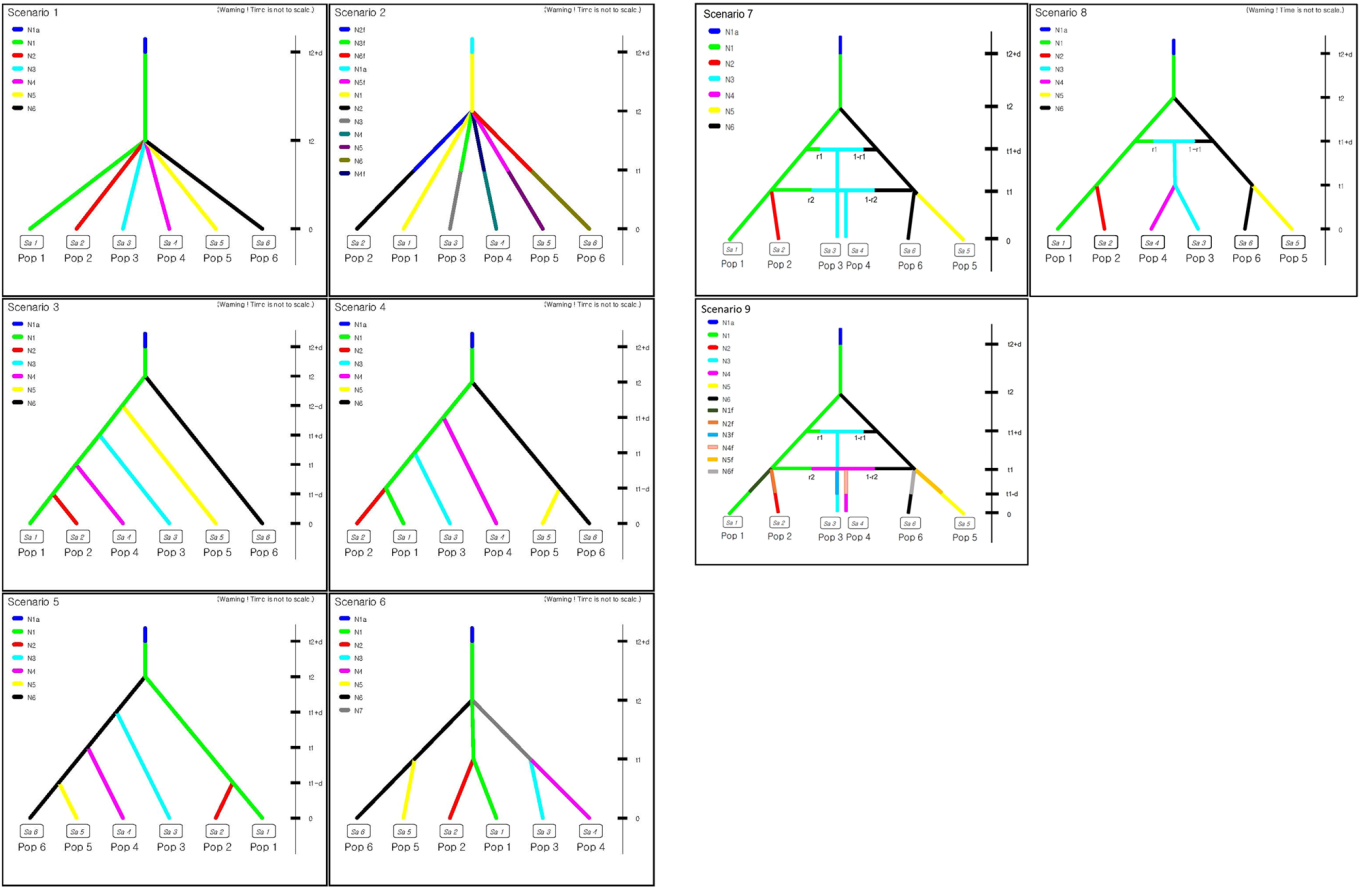
Graphical illustration of the nine evolutionary scenarios of *Asparagus cochinchinensis* examined in the DIYABC analysis. See Table 1 for cluster identifications used in the DIYABC analysis (Pop1-Pop6).

In the DIYABC results, of the nine demographic history models, scenario 7 was the model of choice with the highest posterior probability for both direct and logistic posterior probability estimates, followed by scenario 8 and scenario 4 (Table 5). The best evolutionary model suggested that the two distinct genetic sources (GND, China and TPE, Taiwan) found in the assignment analyses were diversified about 7,000 generations ago from the ancestral population (Table 5; Figs 4 and S5). The Korean and Japanese populations then derived from the GND and TPE nearly 600 generations ago (Table 5; Figs 4 and S5). Of the five Korean populations, two (BOR & HAM) were likely derived from inland China (GND), whereas NAH & YGW originated from Taiwan (TPE; Fig. 4). The two populations (WND & OKN) with intermediate allele frequencies between Taiwanese and Chinese populations were associated with admixture between the two sources (Table 5; Fig. 4). Of the two admixed populations, the Japanese population (OKN) diverged earlier than the Korean admixed population (WND; Table 5; Figs 4 and S5). In the model checking, the summary statistics of the observed data were within the first three PCs estimated from the posterior and prior distributions of the simulated data for scenario 7 (Fig. S4). Also, for the most part, in scenario 7, the summary statistics of observed data did not show significant differences from the ones estimated from the simulated data (Table S3). Type I error of scenario 7 was high (direct measure & logistic measure = ~0.3), indicating that there is a high probability for the chosen scenario to be rejected when it is true. In contrast, Type II errors of scenario 7 from both the direct and logistic estimates were very low (direct measure = ~0.03; logistic measure = ~0.05) providing high confidence with regard to avoiding false positives.

**Table 5:** Summary of the DIYABC analysis.

Scenario	Parameter	Mean	Median	Q0.05	Q0.95	Direct	Logistic
**7**						**0**.**18 [0**.**01**,**0**.**52]**	**0**.**37 [0**.**16**,**0**.**59]**
	N1	1.22E + 03	1.09E + 03	4.25E + 02	2.41E + 03		
	N2	1.29E + 03	7.87E + 02	2.02E + 02	4.49E + 03		
	N3	2.88E + 02	1.35E + 02	4.05E + 01	8.68E + 02		
	N4	2.91E + 03	2.32E + 03	6.16E + 02	7.56E + 03		
	N5	5.47E + 03	5.33E + 03	2.20E + 03	9.08E + 03		
	N6	3.38E + 03	3.09E + 03	1.19E + 03	6.74E + 03		
	t1	6.28E + 02	6.38E + 02	2.94E + 02	9.28E + 02		
	r2	6.09E − 01	6.20E − 01	3.57E − 01	8.20E − 01		
	d	5.99E + 01	6.24E + 01	1.59E + 01	9.68E + 01		
	r1	4.92E − 01	4.92E − 01	2.22E − 01	7.65E − 01		
	t2	6.69E + 03	5.97E + 03	5.06E + 03	1.10E + 04		
	N1a	4.03E + 03	3.50E + 03	8.59E + 02	8.70E + 03		
	µ_cpSSR	1.35E − 04	1.23E − 04	3.15E − 05	6.56E − 05		
	µ_nrSSR	2.06E − 04	1.49E − 04	1.00E − 04	2.06E − 04		
1						0.09 [0.00, 0.34]	0.00 [0.00, 0.16]
2						0.08 [0.00, 0.32]	0.00 [0.00, 0.16]
3						0.03 [0.00, 0.17]	0.00 [0.00, 0.16]
4						0.11 [0.00, 0.38]	0.27 [0.00, 0.57]
5						0.13 [0.00, 0.43]	0.02 [0.00, 0.18]
6						0.07 [0.00, 0.29]	0.00 [0.00, 0.16]
8						0.17 [0.00, 0.49]	0.32 [0.16, 0.49]
9						0.15 [0.00, 0.46]	0.02 [0.00, 0.17]

The direct posterior probability estimates (95% confidence interval) and those calculated by logistic regression for the top 1% of simulated data sets closest to the empirical data for all scenarios are present. For the most probable evolutionary scenario (7), the posterior mean and median values with 95% confidence intervals of parameters integrated in the scenario of choice are provided. The most probable scenario is marked in bold for the posterior probability estimates.

## Discussion

Population divergence is a strong driving force for species range shift and lineage divergence. *Asparagus cochinchinensis* is distributed widely in east Asia, yet the habitat types vary greatly throughout the range. Besides the complex pattern of within and among population-level genetic diversity, our molecular analyses on *A*. *cochinchinensis* provide two major findings. First, our assignment analyses and the ABC model of choice suggest that there are two distinct ESUs (GND, China & TPE, Taiwan) which diverged around LGM and that later each ESU migrated to neighboring regions. Secondly, with the migration (~1,800 BP), the two ESUs were admixed perhaps through secondary contacts independently in Korea (WND) and Japan (OKN).

We found significant departures from HWE in two loci. Heterozygote deficiency observed in one of the two loci likely derived from the presence of null alleles. In contrast, the other locus that deviated from HWE showed an excess of heterozygotes (average observed heterozygosity = 0.75), yet what caused the heterozygote excess is not clear. A few explanations can be considered: small sexual and self-incompatible populations, over dominance, negative assortative mating and asexual reproduction^22^. *A*. *cochinchinensis* is an outcrossing dioecious plant that can clonally propagate through tuberous roots^9,23^. Thus, all four mechanisms are likely causes of heterozygote excess. Unfortunately, with the current data set, we cannot explicitly determine the underlying mechanism of the heterozygote excess for the locus. Future study may further examine factors leading to heterozygote excess for certain loci using both molecular tools and breeding experiments with a variety of sample sizes, as in Stoeckel *et al*.^22^.

We found much lower within-population genetic variation in *A*. *cochinchinensis* (mean He = 0.32; mean Ho = 0.29 Table 1) than in other short-lived perennials estimated from microsatellite data (He = 0.55; Ho = 0.53)^24^. In plants, life history traits and several evolutionary forces (i.e. effective population size (Ne), selection, and demographic changes) influence the level of genetic diversity^25,26^. Since outcrossing plants often show higher within-population genetic diversity^25^, the lowered genetic variation in our results is rather surprising. Inbreeding and clonal reproduction may negatively influence the level of genetic diversity depending on the dispersal and pollination modes, even in dioecious species^27,28^. However, clonal assignment results failed to find significant contribution of clonal reproduction in *A*. *cochinchinensis* collected for the study. We only found a significant inbreeding rate in one population, HAM (F_IS_ = 0.34). However, the genetic diversity in HAM was not the lowest among the eight populations (Table 1). No recent bottleneck events (within the last 3–5 generations) were detected in both the Bottleneck and DIYABC results (Tables 4 and 5). Furthermore, the best evolutionary scenario identified by DIYABC suggested that the Ne estimates are moderate to large (>325; Table 5). One plausible explanation for the reduction in genetic diversity is long-term demographic changes. In fact, the M ratios (G-W index) computed for all eight populations were much lower than the critical value (M = 0.73) estimated using Critical_M, which strongly suggests long-term population bottlenecks^29^.

The average pairwise F_ST_ values of *A*. *cochinchinensis* across all population pairs (0.43; Table 2) were clearly higher than the average F_ST_ values estimated in long-lived perennial plants (microsatellite-based data F_ST_ = 0.31)^24^. In general, outcrossing plants show lower population divergence (e.g. average F_ST_ = 0.22 for 71 outcrossing spp.)^24^, yet *A*. *cochinchinensis* populations exhibited high divergence for an outcrossing species. Coupled with outcrossing nature, long distance dispersal is also likely to alleviate population divergence. Although there is a complete lack of numerical data examined for *A*. *cochinchinensis* thus far, in several congeneric species the most common dispersal vector, birds, can transport the seeds up to 12 km^30,31^. Long distance migration by avian vectors facilitates gene flow among local populations, which genetically homogenizes populations. Despite the dispersal capability and the outcrossing nature of *A*. *cochinchinensis*, we found inflated population divergence, somewhat opposite to what was expected. The migration rates estimated among population pairs also supported the limited gene flow for population pairs. For example, the population pair with the largest F_ST_ (OKN-HAM, F_ST_ = 0.63) had very low migration rate (0.01; Table S2). As shown in the lowered migration rates, limited gene flow among population pairs over long geographic distances might partly be responsible for the large F_ST_ values. This can be supported by our large geographic-scale data collection effort (the longest distance between population pairs, ~2000km). To investigate more closely whether the limited gene flow is associated with geographic distance, we performed a correlation test between genetic divergence and geographic distance (Mantel test).

Notably, we found no significant relationship between genetic divergences and geographic distances. Population pairs within Korea showed greater F_ST_ values (e.g. F_ST_ between HAM and YGW = 0.5) compared to those from population pairs across different nations (F_ST_ between TPE and YGW = 0.1; Table 2). The estimated migration rates also revealed a similar pattern. Despite the long geographic distance, the highest migration rate assessed was between the Chinese population (GND) and the Korean population (BOR; 0.21; Table S2). The F_ST_ values found here are closely associated with the genetically affiliated groups of populations as identified in the STRUCTURE and PCoA results (Table 2; Figs 2 and 3).

AMOVA results from both maternally inherited cpSSRs and nrSSRs were also rather complex for an outcrossing plant, where most of genetic and haplotype variance derived from the group level and, not from the individual level. The complex patterns of genetic variation and population divergence are consistent with the hypothesis of multiple ESUs. As explained by the hypothesis, past and recent species range expansions of divergent lineages accompanied with demographic changes is likely the causal mechanism of the unusual patterns of population divergence. One can argue that the divergence results are due to stochasticity and/or unrealistic assumptions built into the indirect measure of gene flow (F_ST_ estimates), i.e., no mutation, no selection and approximately equal population sizes^32^. To rule out the alternative hypotheses, we examined the spatial population structure among the eight populations through assignment analyses and competed plausible evolutionary hypotheses utilizing the ABC approach.

Consistent with the population divergence pattern, the clustering analyses (PCoA & STRUCTURE) revealed a complex pattern of population structure that does not reflect geographical distances among populations (Figs 2 and 3). Despite the geographic proximity, four Korean *A*. *cochinchinensis* populations were separately allied with two genetically distinct Chinese and Taiwanese population (Figs 2 and 3). One Korean population (WND) was genetically affiliated with the Japanese population, which is geographically more distant than the four remaining Korean populations (Figs 2 and 3). Because the delta K measures showed multiple peaks in association with varying Ks, we presented assignment plots for multiple K values and more closely examined the clustering patterns. The overall divergence pattern dividing the eight populations into two lineages, the Chinese population represented by GND and the Taiwanese population (TPE) remains consistent throughout varying K clusters (K 2 to K 6) with a few exceptions (Fig. S3). For example, K 6 (the cluster number with third highest delta K; Table S4; Fig. S2) revealed the same split pattern of two major clusters as in K 2, but with more complexity. YGW and TPE predominantly consisted of the same cluster (yellow) while BOR and GND shared a different cluster (red; Fig. S2). HAM, however, was assigned to a unique pink cluster. Similarly, NAH was mostly assigned to the blue cluster that is distinct from the YGW cluster pattern. Although the higher K values showed more complex genetic structure patterns, there are a couple of important patterns in common across various K numbers. First, the Chinese and the Taiwanese populations always appeared to be distinct from each other resulting in at least two diverged clusters. The genetic affinities among Korean populations did not mirror the geographic proximity, instead, the Korean populations consisted of the two largely diverged clusters (ESUs).

Cryptic diversity associated with ESUs and/or cryptic species greatly influence the spatial structure among populations. One approach to diagnose the cryptic diversity was to determine if groups share greater genetic affinity across regions than within the same region^1,33^. The clustering pattern observed in our nrSSR data indicated that *A*. *cochinchinensis* in Korean populations likely consists of at least two ESUs with admixture between the two clusters, possibly in secondary contact zones. The clustering results are also congruent with the hypothesis proposed in previous studies based on the distribution and ecological characteristics of the relevant habitats^14,18,34^. In addition, the weak correlation between geographic distance and population divergence from the Mantel test (r = 0.01, P > 0.5; Fig. S1) supports this cryptic diversity pattern.

The divergence pattern observed in our haplotype analysis (cpSSR) differed notably from the pattern found in the nrSSR data. Compared to biparental nuclear markers, maternally transmitted chloroplast markers often show greater population divergence with a high level of variance (near zero to complete fixation) due to various factors, including a seed-only migration mode, the absence of meiotic recombination and increased sensitivity to stochasticity^35,36^. Our cpSSR data showed a rather high level of population divergence with highly inflated heterogeneity (Φ_PT_ values ranging from 0 to 1; Table 2). The Korean populations were not highly diversified, whereas the haplotype divergences were very high between population pairs across Korea and China as well as Korea and Japan. As the cpSSR has much lower evolutionary rates^37^, the pattern of haplotype divergence among populations observed might only be the signature of ancient diversification. The time since demographic events, such as past and recent migrations may not be long enough to accumulate new mutations; thus, the demographic events may not have contributed to the haplotype divergence pattern.

Alternatively, the haplotype divergence pattern may solely stem from random drift, as chloroplast markers are much more sensitive to stochastic events than biparental nuclear markers^36^. Moreover, the pattern might result from sampling bias and/or from the extremely limited variation; thus, extreme caution should be taken when interpreting the pattern. Of 15 haplotypes, most were identified by rare alleles and were in very low frequencies. A single haplotype (12) predominantly occurred in most Korean populations, while the Chinese population (GND) was solely assigned to another single haplotype (7). The Taiwanese population (TPE) and one Korean population (NAH) showed the largest haplotype diversity and shared similar haplotypes (Fig. 1; Table S1).

To shed light on the complex divergence pattern and reconstruct the evolutionary path of *A*. *cochinchinensis* in Korea, we utilized the ABC approach and devised nine final evolutionary scenarios. The most probable scenario (scenario 7) demonstrated that lineage separation between two ancestral populations (GND, China; TPE, Taiwan) dated back to LGM (~21,000 BP; considering an average generation time of three years). This is a crude estimation to some extent, but it is consistent with the major changes in the distribution of the vegetation during LGM throughout east Asia^38^. The ABC results also ruled out the stochasticity hypothesis, as our data did not support the scenario with independent divergence from an ancestor with subsequent founding events (see scenario 2 & scenario 9 in Table 5; low probabilities shown in models associated with founding events in preliminary runs, results not provided). Particularly, scenario 9 was added in the DIYABC analysis to determine if there were recent population bottlenecks after the initial splits, migrations and admixtures set in the scenario 7, the most likely model (Fig. 4; Appendix S1). The probability of scenario 9 was low in the direct measure and zero in the logistic estimation (Table 5). Although our data could not confirm an precise number of ESUs due to limited sampling in China, in the best scenario, there were at least two ESUs in *A*. *cochinchinensis* supporting the hypothesis of ESUs. Each ESU can be an important management unit and should be treated separately for conservation and management practices. Based on the pattern of the admixture between the two ESUs in WND and OKN, there is no complete reproductive barrier between the two ESUs, thus the two ESUs may not be as largely divergent as separate species that are morphologically cryptic.

According to the best model, the Korean populations diverged from the Chinese and Taiwanese populations approximately 1,800 years ago when there were active trading events among the three countries (Korea, China and Japan; Table 5; Fig. 4). The species has been heavily used as a traditional medicine for a long time in Korea. The name of the species and its usages were listed in several ancient medicine books, yet there were no records of natural populations^39^. The evolutionary scenario of choice strongly suggests that Korean *A*. *cochinchinensis* populations were likely introduced from China and Taiwan. The two genetically distinct ESUs, GND (Chinese, Pop1 in the model) and TPE (Taiwanese, Pop6) were independently introduced to the two clusters BOR & HAM (Pop2) and NAH & YGW (Pop5; Figs 2 and 4). The scenario is again consistent with the species introduction hypothesis proposed by distribution and genetic studies^14,18^. The introduction history was also explained by the clustering pattern we found in the PCoA and STRUCTURE results (Figs 2 and 3). Notably, one Korean (WND) and one Japanese (OKN) population shared alleles with both ESUs, perhaps through secondary contact in Korea and Japan.

Scenario 8 with the second highest probability suggested nearly identical demographic history except for the WND admixture event whereas the third best scenario (4) largely differ from the top two scenarios (Fig. 4; Appendix S1). The direct estimate of posterior probability for the scenario was nearly as good as that in our scenario of choice; however, the statistical support was certainly not significant (95% CI overlapping with zero in both the direct and logistic estimates of probability; Table 5). In scenario 4, most of the Korean populations are derivatives of GND except for one group (YGW & NAH, pop 5), which derived from TPE. Scenario 4 showed a high logistic posterior probability (about half that of the scenario of choice), but the direct estimate was nearly zero and the 95% CI completely overlaps with zero. Among five repeated runs with varying parameter sets, we compared two runs showing good shapes of parameter posterior probability-distributions. One of the runs with a broader t1 range had a clear peak for the t1 posterior probability-distribution; however, the run exhibited a poor overall model fit (i.e. the first three PCs in the observed data were not present within the simulated prior distribution). Therefore, we selected the run with a better model fit for further discussion and presented the results here.

Despite its ecological and economical importance as a rare medicinal plant, there is a serious lack of knowledge about the evolutionary features for *A*. *cochinchinensis*. To date, our study is the first empirical study to examine the patterns of genetic diversity across East Asia for the species. Through a genetic diversity analysis, we determined that there are at least two ESUs and inferred the detailed demographic history of *A*. *cochinchinensis* in Korea. The plants have been widely used for several medicinal purposes and farmers started to cultivate the plants in Korea without knowledge of their genetic entities. Our findings of multiple ESUs will provide valuable information to those who cultivate the species. For example, the two distinct ESUs may require different growth conditions. In addition, the two ESUs may have to be closely monitored to prevent losing one of them through hybridization, as found in two locations in Korea and Japan.

## Materials and Methods

### Study species

*Asparagus cochinchinensis* is a dioecious perennial herb in the genus *Asparagus*, subgenus *Asparagus*, a group of dioecious taxa^9^. Sexual differentiation of flowers in *Asparagus* occur in the late stage of development by aborting pistils or stamens selectively, which suggests that the evolution of sex in *Asparagus* was derived from hermaphrodite ancestors^9,40^. The species can be identified from its close relatives by having three cladodes coming from one fascicle^13^. The climbing stems of *A*. *cochinchinensis* grow up to 2 m and are slightly woody^13,23^. The plants flower from May to June and produce green berries with 1–2 black seeds maturing in early fall^13,23^. The fleshy berries attract frugivorous birds as seed disperser in the genus^10,41^. Although studies empirically investigating the reproduction mode of *A*. *cochinchinensis* are lacking, the plant is likely to reach maturity in approximately two to five years for first flowering based on observations made of allied species in the same genus^10^. The species can also clonally grow from the root^34^.

### Sample collection and DNA isolation

In the springs of both 2015 and 2016, we collected young leaves of 158 individuals from eight populations (~20 samples from each population) from Korea, China, Japan and Taiwan (Table 1; Fig. 2). Under the supervision of local botanists, we chose one population confirmed to be a natural population from neighboring countries, China, Japan and Taiwan. Due to over harvesting and private farming, only a small number of natural populations remain in Korea. Among those, we randomly chose five collecting sites with a minimum distance of 50 km between populations (Fig. 2). As *A*. *cochinchinensis* is only distributed south of Chung-Cheong province on the Korean peninsula^14^, there was no need of sampling in North Korea. The plants were identified based on the taxonomic keys formerly described^13,42^. Prior to field sampling, we obtained all required permits from the Ministry of Environment and from the local governments. Two voucher specimens were prepared for each population and were deposited in the National Institute of Natural Resources herbarium (voucher numbers, NIBRVP0000556137- NIBRVP0000556141; NIBRVP0000601487- NIBRVP0000601500). Leaf samples were stored in plastic bags with silica-gel desiccant until DNA extraction. Genomic DNA from dried leaves was isolated using the DNeasy Plant Mini Kit (Qiagen, Hilden, Germany) following the manufacturer’s protocol. We measured the quantity and quality of extracted DNAs in a NanoDrop ND1000 (Thermo Fisher Scientific, Massachusetts, USA; quality cutoff, OD 260/280 ratio between 1.7–1.9) and visualized in 1% agarose-gel electrophoresis. The isolated DNAs then were stored at −20 °C until further use.

### Nuclear and chloroplast microsatellite genotyping

PCR amplification for 13 microsatellite markers (nrSSR) was conducted to genotype all 158 individuals using primer sets developed by Kim *et al*.^21^. PCR reactions were performed in a 25 μl volume containing 2.5 μL of 10× Ex Taq buffer (TaKaRa Bio, Otsu, Japan), 2 μL of 2.5 mM dNTPs, 0.01 μM of each of the forward and reverse primers, 0.1 μL of Ex Taq DNA polymerase (5 units/μL) (TaKaRa Bio), 5–10 ng of template DNA, and distilled water (Sigma-Aldrich Co., St. Louis, Missouri, USA). The PCR cycling conditions were as follows: an initial denaturation step at 98 °C for 5 min followed by 30 cycles of denaturation at 95 °C for 1 min, annealing at 55–57 °C for 1 min, and an extension at 72 °C for 1.5 min, with a final extension step at 72 °C for 10 min. The fluorescently labelled (HEX, FAM) PCR products were pooled with the size standard GS500LIZ (Applied Biosystems, USA) and the amplified fragments were separated out in an ABI 3730XL automated sequencer (Applied Biosystems, USA). Microsatellite profiles were examined on GeneMarker program v. 2.40 (Softgenetics LLC) with automated scoring. The scoring results were manually checked in the final step. We evaluated presence of null alleles and possible scoring errors using FreeNA^43^. Additionally, we amplified chloroplast microsatellites (cpSSR) for 50 individual genotypes subsampled from the total of 158 samples genotyped by nuclear microsatellites using three universal primer pairs^44^ and three primer pairs developed for the allied genus *Maianthemum* in the family, Asparagaceae^45^. Of the six primer pairs, three were successfully amplified and polymorphic at the population level. We assayed genetic variation with the three cpSSRs for all 158 genotypes and used the variation for further analyses.

### Data analysis

Given the possibility of clonal propagation, we first tested for clonality to avoid presence of multiple clones within each population. All 158 samples were assigned to genets based on the 13 nrSSRs in GenoDive v. 2.0b23^46^. To discriminate true clones from clones produced through sexual reproduction by chance, the probability of identical MLG due to sexual reproduction by chance was calculated using the binomial probability function Psex^47^. Because there were only a few identical clones, we did not account for clonality in the subsequent analyses. We estimated the genetic diversity parameters; He, Ho, Na and F_IS_ using Arlequin v. 3.5 and GENALEX v. 6.502^48,49^. We tested for significant deviation from HWE and LD in 13 microsatellite loci within each population using Fisher’s exact test^50^ in Arlequin. Bonferroni corrections for multiple comparisons (adjusted P values) were applied. Four of thirteen microsatellite markers significantly deviated from HWE and/or were not independent of the remaining markers. Certain genetic analyses, such as STRUCTURE and ABC assume HWE and/or independence among markers. To avoid bias influenced by the number of markers used between different analyses, we screened out those 4 markers from all downstream analyses. We calculated pairwise F_ST_ between all population pairs in Arlequin with 1,000 permutations for the significant alpha. We tested for Isolation by Distance based on the pairwise genetic distance (Slatkin’s linearized F_ST_ = F_ST_/(1 − F_ST_)) and log-transformed Euclidean distance for all population pairs in GENALEX^49,51^. For statistical significance of the correlation coefficient (r), 1,000 random permutations with replacement were used.

Haplotype diversity of the cpSSRs was assessed as the total number of haplotypes (Nh), haplotype diversity (Hehap) and pairwise genetic differentiation among populations (Φ_PT_), and the analogue of GST for haplotype data (Nei’s coefficient of gene differentiation; Nei, 1973) in GENALEX^49^. We used 1,000 permutations with replacements for the significance tests. The hierarchical distribution of haplotype variation among four regional groups (Korea, China, Taiwan, and Japan) was evaluated by analysis of molecular variance (AMOVA) implemented in Arlequin^48^. Given the limited variation found in our haplotype analysis, we examined genetic structures only by plotting the distributions of haplotypes using a median-joining network in Network 5 (http://www.fluxus-engineering. com). Alleles were recoded as size of fragments, where 1-bp size change was given a weight of 1^52^.

Population structures was examined using a Bayesian model-based clustering approach with the correlated allele frequencies model^53^ implemented in STRUCTURE v. 2.3.4^54^. As described above, we screened out four microsatellite markers that were violating the assumptions of panmixia and independence among markers and used the admixture model. Ten repeats of independent runs were conducted with 1,000,000 MCMC iterations following 100,000 steps as burn-in for each K from 1 to 8. We inferred the optimal number of clusters, K based on ΔK as estimated following Evanno *et al*.^55^ in STRUCTURE HARVESTER v. 0.6.94^56^. We used CLUMPP v. 1.1.2 to summarize the individual ancestry coefficients from 10 STRUCTURE repeats with the greedy option^57^. The summary of the results was visualized in DISTRUCT v. 1.1^58^. We also performed a Principal Coordinate Analysis (PCoA) on the pairwise Nei’s genetic distance estimated for all 158 individuals using GENALEX. AMOVA was used to partition molecular variance hierarchically within and between clusters defined from both STRUCTURE and PCoA in Arlequin. We compared the estimated values against values calculated from 1,000 resampled data sets for statistical significance.

We examined reduction in population size derived by historical and recent bottlenecks using the Garza-Williamson index (G-W index; M-ratio) implemented in Arlequin and BOTTLENECK v. 1.2.02^59,60^, respectively. Because bottleneck events are assumed to reduce the allele number more rapidly than the allele size ranges, the M-ratio test uses the ratio between the number of alleles and the allele size range^60^. BOTTLENECK detects recent population decline within a few generations by finding a significant excess or deficit of heterozygosity relative to an equilibrium state^61^. A simulation study revealed that the M-ratio is better suited for identifying a historical bottleneck than BOTTLENECK^29^. We ran BOTTLENECK under the infinite allele model (IAM) and stepwise mutation model (SMM) with Sign and Wilcoxon’s sign rank tests for statistical significance. We also estimated the proportion of migrants among the eight populations within the recent past (the last few generations) using BAYESASS 3.0.1^62^. We used 10,000,000 MCMC iterations with a 1,000,000 burn-in and, then sampled every 2,000 generations during the process. The default settings were used for the mixing parameters.

The most probable evolutionary history of *A*. *cochinchinensis* in Korea was determined through the ABC model approach implemented in DIYABC v. 2.1.0^63^. For the ABC computation, we used all 12 microsatellite loci by combining nine nrSSRs and three cpSSRs. Using both cytoplasmic and nuclear molecular data would allow more insights into admixture among genetically distinct lineages. We generated nine likely evolutionary scenarios that summarize the divergence and admixture history among the five Korean populations and the three populations of neighboring countries. (Fig. 4). We formulated a “ghost” population to account for an un-sampled genetic source that might have contributed to divergence patterns in the Korean populations^64^. To simplify the models and ease computational challenges, we delimited the populations to 6 clusters based on the clustering pattern resulting from STRUCTURE, PCoA and the geographic data. Prior to the final nine scenarios, two preliminary DIYABC runs with various numbers of scenarios were conducted to finalize the scenarios to be investigated. We eliminated scenarios that were not biologically justified and chose the final nine plausible evolutionary scenarios (see Appendix S1 for detailed descriptions of the scenarios).

For the nine nrSSRs, we used the default priors of the mutation rate, whereas the prior values of the mutation rate for the three cpSSRs were changed to achieve better posterior distributions (Table S5). Given much lower mutation rates of cpSSRs than nrSSRs as estimated from former studies^37,65^, we scaled the mutation rate of the cpSSRs to be low (Table S5). We used the following summary statistics for each population: mean expected heterozygosity, mean allele size variation and mean number of alleles. For two sample summary statistics, F_ST_, the classification index and the genetic distance were used. We set uniform priors on all demographic parameters (Table S63). As LGM strongly influenced the current distribution of plant species in north east Asia^38^, the historical divergence time (t2) for the species might have dated back to as early as 21,000 years using a generation time of three years (see Vivian-Smith & Gosper^10^ for the generation time). To retain a clear signature for the posterior distribution of each parameter, we repeated the DIYABC runs five times with different mutation models and parameter sets, particularly for divergence times (t1 and t2) with the finalized nine scenarios. A simulation of each scenario was performed for 3 × 10^6^ iterations based on neutral coalescence.

We determined the most probable scenario based on posterior supports of each scenario estimated by two approaches: (1) direct estimates by the frequency of a given scenario with the 500 data sets generating summary statistics that most closely matched the observed data and (2) logistic regression^63^. We determined the demographic parameters of the most probable scenario from 10,000 simulated datasets closest to the observed data. We assessed the goodness-of-fit for each model by the Principal Component Analysis (PCA) approach implemented in DIYABC using by model checking function. The function evaluates the discrepancies between the simulated and the observed data. To determine the level of confidence of the chosen scenario, we estimated Type I and Type II errors using the confidence option in DIYABC with a simulation of 500 datasets. Type I error for scenario 7, the scenario of choice, was estimated as the probability at which scenario 7 is rejected when it is the true scenario. Type II error was calculated as the probability of choosing a scenario when it is not the true scenario.

## Supplementary information

Supplementary information

## Data Availability

Microsatellite data are available in the DRYAD Digital Repository (10.5061/dryad.k10p97v).
