# Supplementary material for: An unexpected genetic diversity pattern and a complex demographic history of a rare medicinal herb, Chinese asparagus (Asparagus cochinchinensis) in Korea
Source: Sci Rep. 2019 Jul 5;9:9757. doi: 10.1038/s41598-019-46275-9 (PMC6611897; doi:10.1038/s41598-019-46275-9)
Supplement: Supplementary file 1 — Supplementary information [file 41598_2019_46275_MOESM1_ESM.docx]

Supplementary Information

Unexpected genetic diversity pattern and a complex demographic history of a rare medicinal herb, Chinese asparagus (*Asparagus cochinchinensis*) in Korea

Authors: Soo-Rang Lee^1^, Han-Sol Park^3^, Bo-Yun Kim^1^, Jung-Hoon Lee^2^, Qiang Fan^4^, John F. Gaskin^5^ and Young-Dong Kim^*2^

^------------------------------------------------------------------------------------------------^

^1^Multidisciplinary Genome Institute, Life Science Hall, Hallym University, Hallymdaehak-gil, Chuncheon-si, Gangwon-do, S Korea 24252

^2^Department of Life Sciences, Life Science Hall # 8311, Hallym University, Hallymdaehak-gil, Chuncheon-si, Gangwon-do, S Korea 24252

^3^National Institute of Biological Resources, 42 Hwangyeong-ro, Seo-gu, Incheon 22689 S Korea

^4^State Key Laboratory of Biocontrol and Guangdong Provincial Key Laboratory of Plant Resources, Sun Yat-sen University, Guangzhou, China 510275

^5^ USDA-ARS, 1500 North Central Avenue, Sidney, Montana USA 59270

Table S1. Fifteen distinct cpSSR haplotypes identified from 158 *Asparagus cochinchinensis* samples of eight populations.

| Sample | Populations | Haplotype Code |
| --- | --- | --- |
| 2015ASP001 - 2015ASP012 | YGW | 12 |
| 2015ASP013 | YGW | 14 |
| 2015ASP014 | YGW | 12 |
| 2015ASP015 | YGW | 15 |
| 2015ASP016 - 2015ASP020 | YGW | 12 |
| 2015ASP026 - 2015ASP045 | HAM | 12 |
| 2015ASP095 - 2015ASP101 | WND | 12 |
| 2015ASP102 | WND | 2 |
| 2015ASP103 - 2015ASP110 | WND | 12 |
| 2015ASP111 | WND | 9 |
| 2015ASP112 | WND | 12 |
| 2016ASP043 - 2016ASP047 | BOR | 12 |
| 2016ASP048 - 2016ASP062 | BOR | 13 |
| 2016ASP049 - 2016ASP062 | BOR | 12 |
| 2016ASP067 - 2016ASP072 | NAH | 12 |
| 2016ASP073 - 2016ASP074 | NAH | 13 |
| 2016ASP075 | NAH | 12 |
| 2016ASP076 | NAH | 10 |
| 2016ASP077 | NAH | 12 |
| 2016ASP078 | NAH | 11 |
| 2016ASP079 - 2016ASP080 | NAH | 12 |
| 2016ASP081 - 2016ASP084 | NAH | 13 |
| 2016ASP085 | NAH | 10 |
| 2016ASP086 | NAH | 12 |
| 2015ASP049 | TPE | 7 |
| 2015ASP050 | TPE | 12 |
| 2015ASP051 | TPE | 8 |
| 2015ASP052 | TPE | 13 |
| 2015ASP053 | TPE | 3 |
| 2015ASP055 | TPE | 3 |
| 2015ASP056 | TPE | 4 |
| 2015ASP057 | TPE | 1 |
| 2015ASP058 | TPE | 12 |
| 2015ASP059 - 2015ASP060 | TPE | 4 |
| 2015ASP061 - 2015ASP062 | TPE | 7 |
| 2015ASP063 - 2015ASP064 | TPE | 12 |
| 2015ASP065 - 2015ASP066 | TPE | 7 |
| 2015ASP067 2015ASP069 | TPE | 12 |
| 2016ASP001 - 2016ASP002 | OKN | 6 |
| 2016ASP003 - 2016ASP006 | OKN | 7 |
| 2016ASP004 - 2016ASP006 | OKN | 6 |
| 2016ASP007 - 2016ASP009 | OKN | 7 |
| 2016ASP010 | OKN | 5 |
| 2016ASP011 | OKN | 7 |
| 2016ASP012 - 2016ASP020 | OKN | 6 |
| 2016ASP023 - 2016ASP042 | GND | 7 |

Table S2. Contemporary migration rates between population pairs estimated from BAYESASS. See Table 1 for population abbreviation. CI stands for confidence interval. M_F_ indicates migration rates of one direction and M_R_ represents the migration rate of reverse direction for the same population pair.

| Group1 | Group2 | Mf [±95%CI] | Group1 | Group2 | Mr [±95%CI] |
| --- | --- | --- | --- | --- | --- |
| HAM | YGW | 0.0121 [0.0116] | YGW | HAM | 0.0119 [0.0115] |
| WND | YGW | 0.0136 [0.0130] | YGW | WND | 0.0124 [0.0120] |
| BOR | YGW | 0.0119 [0.0116] | YGW | BOR | 0.0119 [0.0115] |
| NAH | YGW | 0.0126 [0.0122] | YGW | NAH | 0.0128 [0.0124] |
| TPE | YGW | 0.1958 [0.0487] | YGW | TPE | 0.0195 [0.0228] |
| OKN | YGW | 0.0119 [0.0115] | YGW | OKN | 0.0119 [0.0115] |
| GND | YGW | 0.0120 [0.0116] | YGW | GND | 0.0119 [0.0116] |
| WND | HAM | 0.0137 [0.0133] | HAM | WND | 0.0164 [0.0147] |
| BOR | HAM | 0.0458 [0.0343] | HAM | BOR | 0.0204 [0.0196] |
| NAH | HAM | 0.0133 [0.0126] | HAM | NAH | 0.0122 [0.0118] |
| TPE | HAM | 0.0120 [0.0116] | HAM | TPE | 0.0120 [0.0116] |
| OKN | HAM | 0.0120 [0.0116] | HAM | OKN | 0.0122 [0.0117] |
| GND | HAM | 0.0206 [0.0194] | HAM | GND | 0.0120 [0.0116] |
| BOR | WND | 0.0120 [0.0116] | WND | BOR | 0.0133 [0.0128] |
| NAH | WND | 0.0295 [0.0233] | WND | NAH | 0.0311 [0.0214] |
| TPE | WND | 0.0123 [0.0119] | WND | TPE | 0.0132 [0.0126] |
| OKN | WND | 0.0121 [0.0116] | WND | OKN | 0.0132 [0.0129] |
| GND | WND | 0.0154 [0.0145] | WND | GND | 0.0131 [0.0127] |
| NAH | BOR | 0.0123 [0.0118] | BOR | NAH | 0.0119 [0.0115] |
| TPE | BOR | 0.0120 [0.0115] | BOR | TPE | 0.0119 [0.0113] |
| OKN | BOR | 0.0120 [0.0117] | BOR | OKN | 0.0119 [0.0114] |
| GND | BOR | 0.2143 [0.0359] | BOR | GND | 0.0128 [0.0122] |
| TPE | NAH | 0.0121 [0.0118] | NAH | TPE | 0.0123 [0.0118] |
| OKN | NAH | 0.0119 [0.0115] | NAH | OKN | 0.0134 [0.0131] |
| GND | NAH | 0.0121 [0.0117] | NAH | GND | 0.0120 [0.0116] |
| OKN | TPE | 0.0118 [0.0114] | TPE | OKN | 0.0120 [0.0116] |
| GND | TPE | 0.0120 [0.0116] | TPE | GND | 0.0119 [0.0114] |
| GND | OKN | 0.0272 [0.0189] | OKN | GND | 0.0118 [0.0114] |

Table S3. Summary of model checking results. The observed values of summary statistics used and the deviation of observed data from the posterior predictive distribution of simulated data under the most likely scenario (7). NAK= mean number of alleles, HET= mean gene diversity, VAR= mean allele size variance, FST= pair-wise population differentiation, LIK= two sample, mean index of classification and DM= genetic distance. Parameters with _1 are estimated with the three cpSSR loci and parameters with _2 are estimated with the nine nrSSR loci.

| Summary statistics | Observed value | P (simulated<observed) | Significance |
| --- | --- | --- | --- |
| NAL_1_1 | 1 | 0.1285 |  |
| NAL_1_2 | 1.3333 | 0.449 |  |
| NAL_1_3 | 1.6667 | 0.9335 |  |
| NAL_1_4 | 1.6667 | 0.3135 |  |
| NAL_1_5 | 2.6667 | 0.5855 |  |
| NAL_1_6 | 2.6667 | 0.766 |  |
| HET_1_1 | 0 | 0.1285 |  |
| HET_1_2 | 0.0167 | 0.3585 |  |
| HET_1_3 | 0.1561 | 0.8985 |  |
| HET_1_4 | 0.0741 | 0.1685 |  |
| HET_1_5 | 0.1282 | 0.1785 |  |
| HET_1_6 | 0.3825 | 0.719 |  |
| VAR_1_1 | 0 | 0.1285 |  |
| VAR_1_2 | 0.0021 | 0.333 |  |
| VAR_1_3 | 0.6428 | 0.985 | * |
| VAR_1_4 | 0.0231 | 0.117 |  |
| VAR_1_5 | 0.066 | 0.1525 |  |
| VAR_1_6 | 1.6787 | 0.921 |  |
| FST_1_1&2 | 0.9675 | 0.981 | * |
| FST_1_1&3 | 0.6877 | 0.397 |  |
| FST_1_1&4 | 0.9001 | 0.9715 | * |
| FST_1_1&5 | 0.7865 | 0.7925 |  |
| FST_1_1&6 | 0.3961 | 0.1165 |  |
| FST_1_2&3 | 0.8893 | 0.6665 |  |
| FST_1_2&4 | 0.0255 | 0.0765 |  |
| FST_1_2&5 | 0.015 | 0.014 | * |
| FST_1_2&6 | 0.3599 | 0.071 |  |
| FST_1_3&4 | 0.8056 | 0.7855 |  |
| FST_1_3&5 | 0.7703 | 0.8105 |  |
| FST_1_3&6 | 0.4788 | 0.313 |  |
| FST_1_4&5 | -0.0012 | 0.019 | * |
| FST_1_4&6 | 0.2113 | 0.205 |  |
| FST_1_5&6 | 0.2198 | 0.68 |  |
| LIK_1_1&2 | 0.8061 | 0.777 |  |
| LIK_1_1&3 | 0.2122 | 0.2025 |  |
| LIK_1_1&4 | 0.7059 | 0.704 |  |
| LIK_1_1&5 | 0.825 | 0.2535 |  |
| LIK_1_1&6 | 0.2275 | 0.0635 |  |
| LIK_1_2&1 | 0.7269 | 0.797 |  |
| LIK_1_2&3 | 0.9175 | 0.5265 |  |
| LIK_1_2&4 | 0.0505 | 0.08 |  |
| LIK_1_2&5 | 0.0472 | 0.025 | * |
| LIK_1_2&6 | 0.175 | 0.057 |  |
| LIK_1_3&1 | 0.4939 | 0.334 |  |
| LIK_1_3&2 | 1.3551 | 0.636 |  |
| LIK_1_3&4 | 1.1717 | 0.79 |  |
| LIK_1_3&5 | 1.2023 | 0.71 |  |
| LIK_1_3&6 | 0.6807 | 0.455 |  |
| LIK_1_4&1 | 0.7481 | 0.557 |  |
| LIK_1_4&2 | 0.0989 | 0.093 |  |
| LIK_1_4&3 | 0.9435 | 0.503 |  |
| LIK_1_4&5 | 0.1244 | 0.0565 |  |
| LIK_1_4&6 | 0.239 | 0.102 |  |
| LIK_1_5&1 | 0.808 | 0.2035 |  |
| LIK_1_5&2 | 0.1459 | 0.027 | * |
| LIK_1_5&3 | 0.9693 | 0.474 |  |
| LIK_1_5&4 | 0.1628 | 0.07 |  |
| LIK_1_5&6 | 0.2618 | 0.3035 |  |
| LIK_1_6&1 | 0.6634 | 0.139 |  |
| LIK_1_6&2 | 0.587 | 0.084 |  |
| LIK_1_6&3 | 0.8197 | 0.405 |  |
| LIK_1_6&4 | 0.5604 | 0.3545 |  |
| LIK_1_6&5 | 0.5789 | 0.7225 |  |
| DM2_1_1&2 | 5.3334 | 0.9855 | * |
| DM2_1_1&3 | 0.1519 | 0.271 |  |
| DM2_1_1&4 | 5.1865 | 0.923 |  |
| DM2_1_1&5 | 5.269 | 0.722 |  |
| DM2_1_1&6 | 0.8844 | 0.295 |  |
| DM2_1_2&3 | 5.4909 | 0.822 |  |
| DM2_1_2&4 | 0.0013 | 0.05 | * |
| DM2_1_2&5 | 0.0021 | 0.02 | * |
| DM2_1_2&6 | 1.8836 | 0.475 |  |
| DM2_1_3&4 | 5.3383 | 0.864 |  |
| DM2_1_3&5 | 5.4321 | 0.849 |  |
| DM2_1_3&6 | 1.0812 | 0.553 |  |
| DM2_1_4&5 | 0.004 | 0.0355 | * |
| DM2_1_4&6 | 1.7968 | 0.658 |  |
| DM2_1_5&6 | 1.8479 | 0.948 |  |
| NAL_2_1 | 2.5556 | 0.573 |  |
| NAL_2_2 | 1.8889 | 0.238 |  |
| NAL_2_3 | 1.5556 | 0.6535 |  |
| NAL_2_4 | 1.8889 | 0.015 | * |
| NAL_2_5 | 2.7778 | 0.0125 | * |
| NAL_2_6 | 2.2222 | 0.027 | * |
| HET_2_1 | 0.3048 | 0.5 |  |
| HET_2_2 | 0.1945 | 0.2805 |  |
| HET_2_3 | 0.1023 | 0.5245 |  |
| HET_2_4 | 0.2314 | 0.0165 | * |
| HET_2_5 | 0.2812 | 0.0155 | * |
| HET_2_6 | 0.236 | 0.0215 | * |
| VAR_2_1 | 1.4954 | 0.8555 |  |
| VAR_2_2 | 0.8581 | 0.774 |  |
| VAR_2_3 | 0.1791 | 0.574 |  |
| VAR_2_4 | 0.9 | 0.2135 |  |
| VAR_2_5 | 1.2364 | 0.368 |  |
| VAR_2_6 | 0.954 | 0.2975 |  |
| FST_2_1&2 | 0.2044 | 0.4315 |  |
| FST_2_1&3 | 0.5415 | 0.3275 |  |
| FST_2_1&4 | 0.3144 | 0.7785 |  |
| FST_2_1&5 | 0.4005 | 0.428 |  |
| FST_2_1&6 | 0.4503 | 0.478 |  |
| FST_2_2&3 | 0.555 | 0.354 |  |
| FST_2_2&4 | 0.4341 | 0.8375 |  |
| FST_2_2&5 | 0.3987 | 0.261 |  |
| FST_2_2&6 | 0.5047 | 0.462 |  |
| FST_2_3&4 | 0.5131 | 0.5695 |  |
| FST_2_3&5 | 0.44 | 0.476 |  |
| FST_2_3&6 | 0.5722 | 0.6955 |  |
| FST_2_4&5 | 0.2559 | 0.577 |  |
| FST_2_4&6 | 0.3524 | 0.8365 |  |
| FST_2_5&6 | 0.1584 | 0.972 | * |
| LIK_2_1&2 | 0.6896 | 0.305 |  |
| LIK_2_1&3 | 1.2721 | 0.145 |  |
| LIK_2_1&4 | 0.9309 | 0.4835 |  |
| LIK_2_1&5 | 1.26 | 0.028 | * |
| LIK_2_1&6 | 1.3402 | 0.0565 |  |
| LIK_2_2&1 | 0.4809 | 0.142 |  |
| LIK_2_2&3 | 1.0307 | 0.0695 |  |
| LIK_2_2&4 | 0.7949 | 0.326 |  |
| LIK_2_2&5 | 0.9487 | 0.005 | ** |
| LIK_2_2&6 | 1.1289 | 0.0175 | * |
| LIK_2_3&1 | 0.7719 | 0.054 |  |
| LIK_2_3&2 | 1.0728 | 0.104 |  |
| LIK_2_3&4 | 0.7272 | 0.0565 |  |
| LIK_2_3&5 | 1.0038 | 0.102 |  |
| LIK_2_3&6 | 1.1233 | 0.182 |  |
| LIK_2_4&1 | 0.6863 | 0.023 | * |
| LIK_2_4&2 | 0.8282 | 0.038 | * |
| LIK_2_4&3 | 0.9649 | 0.015 | * |
| LIK_2_4&5 | 0.6959 | 0.002 | ** |
| LIK_2_4&6 | 0.8632 | 0.009 | ** |
| LIK_2_5&1 | 1.066 | 0.001 | *** |
| LIK_2_5&2 | 1.0406 | 0 | *** |
| LIK_2_5&3 | 1.1697 | 0.019 | * |
| LIK_2_5&4 | 0.7307 | 0.0075 | ** |
| LIK_2_5&6 | 0.6314 | 0.059 |  |
| LIK_2_6&1 | 1.1495 | 0.002 | ** |
| LIK_2_6&2 | 1.313 | 0.003 | ** |
| LIK_2_6&3 | 1.2845 | 0.033 | * |
| LIK_2_6&4 | 0.9425 | 0.057 |  |
| LIK_2_6&5 | 0.532 | 0.049 | * |
| DM2_2_1&2 | 0.2947 | 0.514 |  |
| DM2_2_1&3 | 0.6459 | 0.1265 |  |
| DM2_2_1&4 | 0.2341 | 0.1125 |  |
| DM2_2_1&5 | 1.711 | 0.1805 |  |
| DM2_2_1&6 | 0.4827 | 0.0115 | * |
| DM2_2_2&3 | 0.7679 | 0.157 |  |
| DM2_2_2&4 | 0.308 | 0.1505 |  |
| DM2_2_2&5 | 1.7808 | 0.192 |  |
| DM2_2_2&6 | 0.4574 | 0.012 | * |
| DM2_2_3&4 | 0.5924 | 0.1155 |  |
| DM2_2_3&5 | 2.8892 | 0.593 |  |
| DM2_2_3&6 | 0.9008 | 0.153 |  |
| DM2_2_4&5 | 1.4848 | 0.454 |  |
| DM2_2_4&6 | 0.2951 | 0.0565 |  |
| DM2_2_5&6 | 0.9303 | 0.897 |  |

Table S4. Probability of each K estimated from 10 repeated STRUCTURE runs summarized as in Evanno et al. (2005). Mean likelihood of each K, LnP(K), standard deviation and delta K are presented.

| K | Repeat number | Mean LnP(K) | Stdev LnP(K) | Delta K |
| --- | --- | --- | --- | --- |
| 1 | 10 | -1855.14 | 0.295146 | — |
| 2 | 10 | -1560.84 | 0.445222 | 237.11791 |
| 3 | 10 | -1372.11 | 0.59339 | 137.27914 |
| 4 | 10 | -1264.84 | 0.414193 | 146.45359 |
| 5 | 10 | -1218.23 | 12.964828 | 1.530294 |
| 6 | 10 | -1151.78 | 1.213626 | 44.601867 |
| 7 | 10 | -1139.46 | 2.081239 | 0.43724 |
| 8 | 10 | -1126.23 | 1.498926 | — |

Table S5. Mutation models and summary statistics incorporated in the ABC analysis.

|  |  | Prior distribution | Mininum | Maximum | Mean | Shape |
| --- | --- | --- | --- | --- | --- | --- |
| cpSSR | Mean mutation rate | Uniform | 1.00E-05 | 5.00E-04 |  |  |
|  | Individual locus mutation rate | Gamma | 1.00E-06 | 1.00E-03 | mean_u | 2 |
|  | Mean coefficient P | Uniform | 1.00E-01 | 3.00E-01 |  |  |
|  | Individual locus coefficient P | Gamma | 1.00E-02 | 9.00E-01 | mean_p | 2 |
| nrSSR | Mean mutation rate | Uniform | 1.00E-04 | 1.00E-03 |  |  |
|  | Individual locus mutation rate | Gamma | 1.00E-05 | 1.00E-02 | mean_u | 2 |
|  | Mean coefficient P | Uniform | 1.00E-01 | 3.00E-01 |  |  |
|  | Individual locus coefficient P | Gamma | 1.00E-02 | 9.00E-01 | mean_p | 2 |

Table S6. Prior settings and descriptions of parameters used to simulate five evolutionary scenarios for 6 *A. cochinchinensis* groups. The population size used is the effective population size (Ne). The unit of time parameters (t1-t2; d) is the generation time of 3 years considering average time of first flowering from seed germination.

| Parameter | Description | Distribution | Min | Max |
| --- | --- | --- | --- | --- |
| N1a | Ancestral population size | Uniform | 10 | 10,000 |
| N1-N6 | Population size of 6 groups defined based on geography and the genetic groups identified by STRUCTURE (Table 1 for population abbreviation) | Uniform | 10 | 10,000 |
| N7 | Population size of an unsampled ghost region | Uniform | 10 | 10,000 |
| N1f-N6f | Founding population size for the defined groups | Uniform | 10 | 10,000 |
| t2 | Early divergence time | Uniform | 2,000 | 15,000 |
| t1 | Time of recent event such as split and/or admixture | Uniform | 20 | 1,000 |
| d | Advanced and/or retreated time difference from the time of recent event | Uniform | 10 | 50 |
| r1&r2 | Rate of admixture | Uniform | 1.00E-03 | 9.99E-01 |

Figure S1: The association between the Euclidean distances log transformed and Slatkin’s linearized F_ST_ (F_ST_ / (1- F_ST_)) for all possible population pairs of 8 *A*. *cocohinchinensis* populations. A Mantel test revealed that there is a complete lack of Isolation by Distance pattern (r=0.014, P > 0.5).


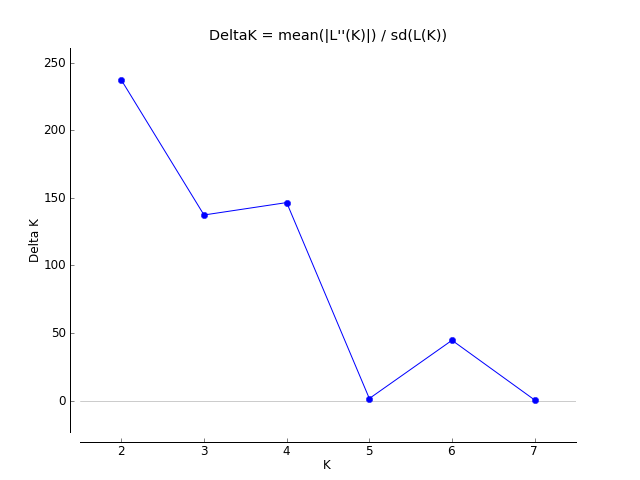


Figure S2: Delta K plots. Based on the STRUCTURE run for 8 *Asparagus cocohinchinensis* populations with 9 microsatellite markers, delta K values for each of K clusters from 2 to 7 were estimated by method of Evanno et al. (2005).


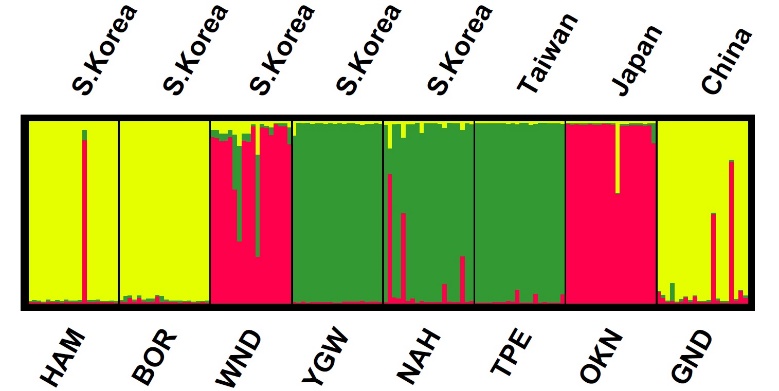
K3


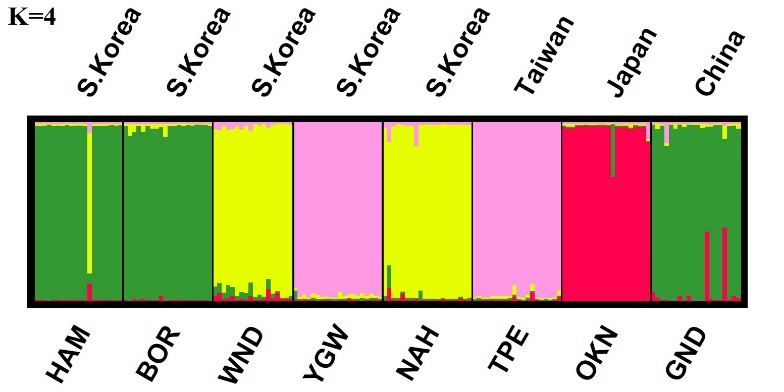
K4


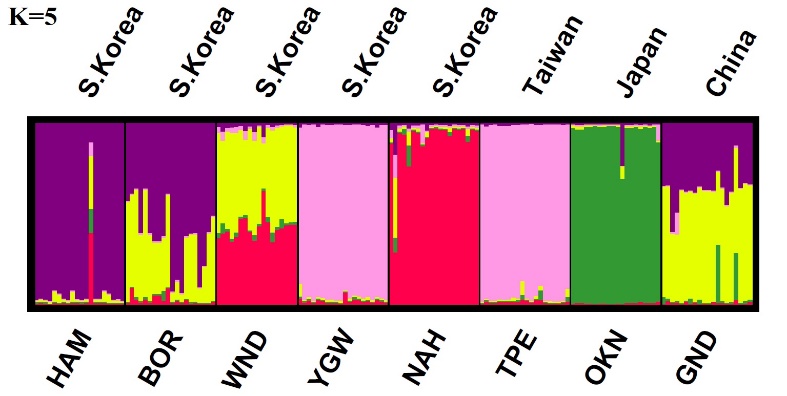
K5


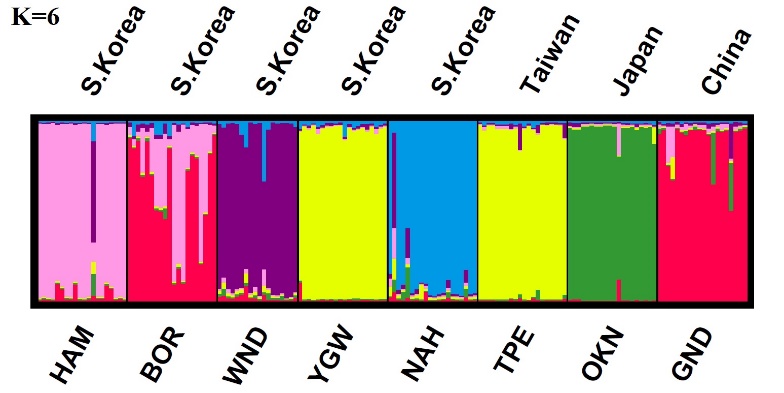
K6


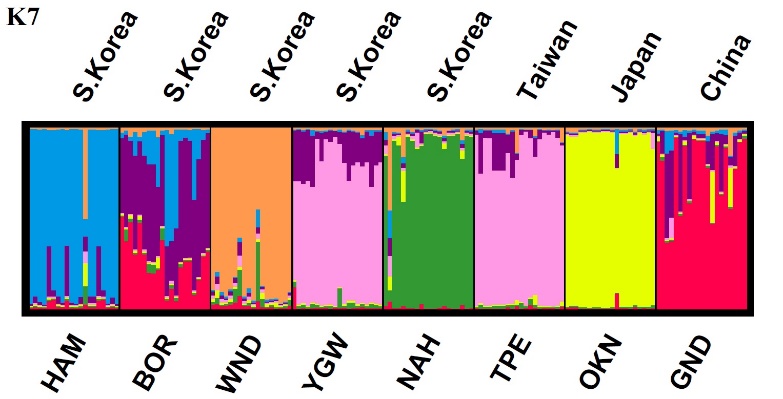
K7


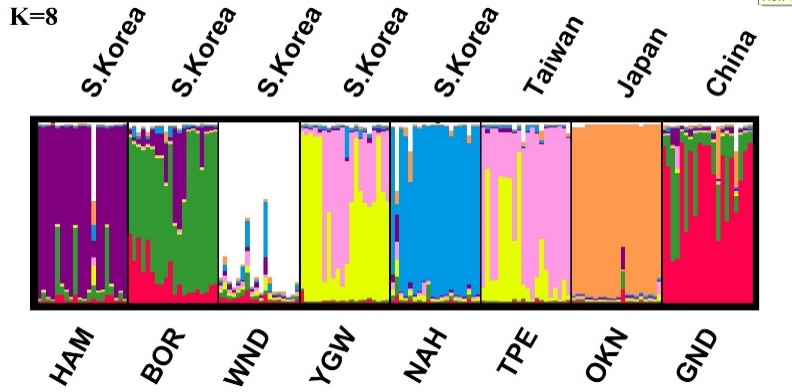
K8

Figure S3: Bar plots of the Bayesian model-based group assignment for the *K* = 2 to *K* = 8 from STRUCTURE run for 8 *A*. *cocohinchinensis* populations with 9 microsatellite markers. Populations are separated by solid vertical black lines. Colors represent assignments of loci into each of the 2-8 estimated groups. See Table 1 for population abbreviations.


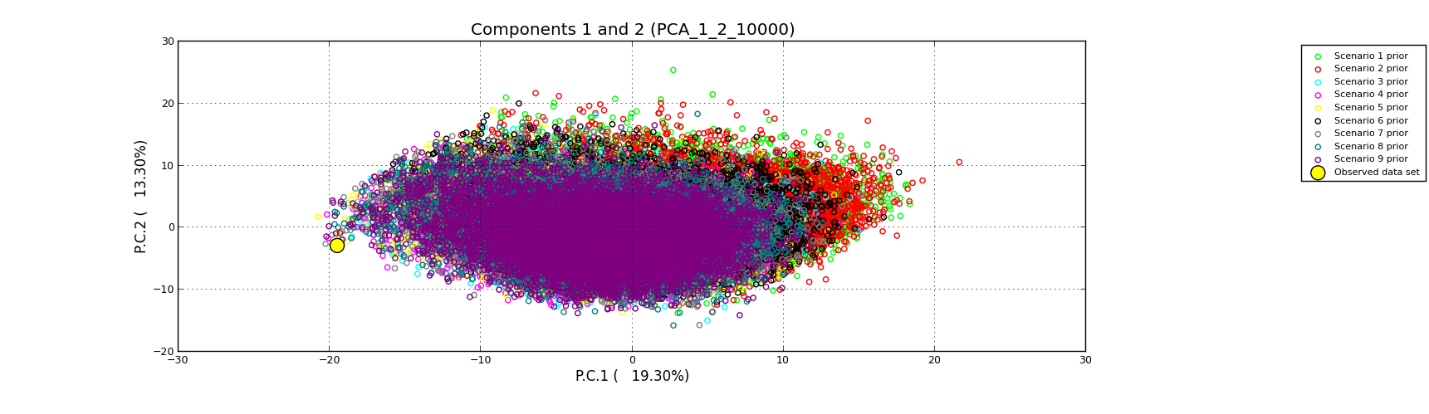


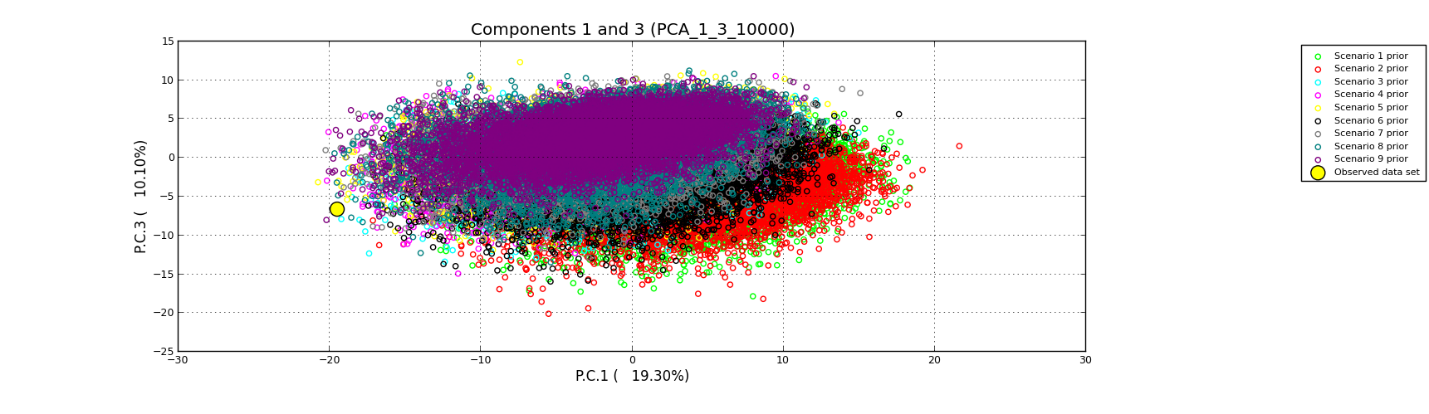


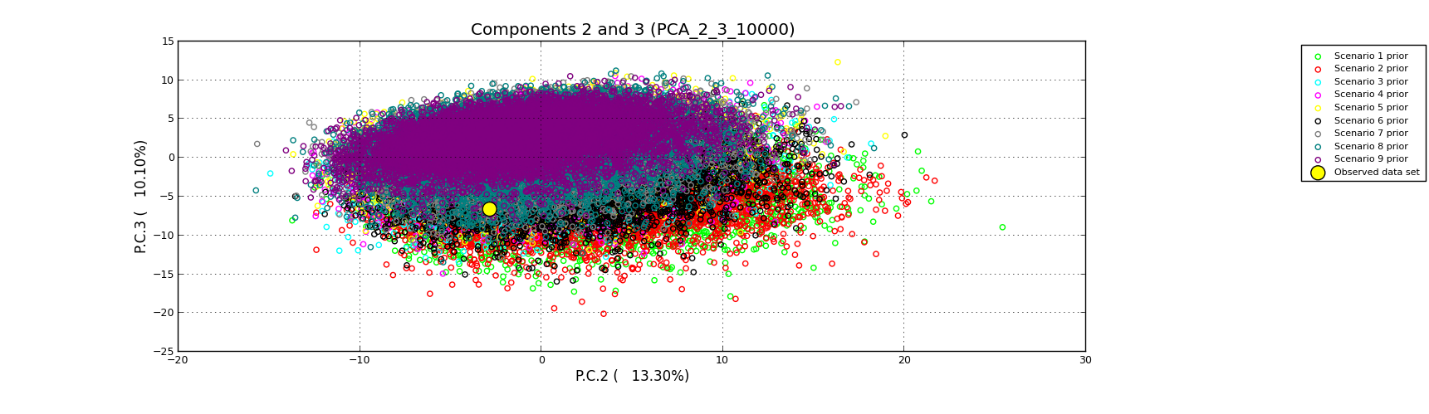


Figure S4. PCA plots for DIYABC model checking. The observed data (yellow circle) were within the first three axes of PCAs on summary statistics estimated from the posterior predictive distribution of parameters (grey circle filled) and simulated prior distribution of parameters (open grey circle).


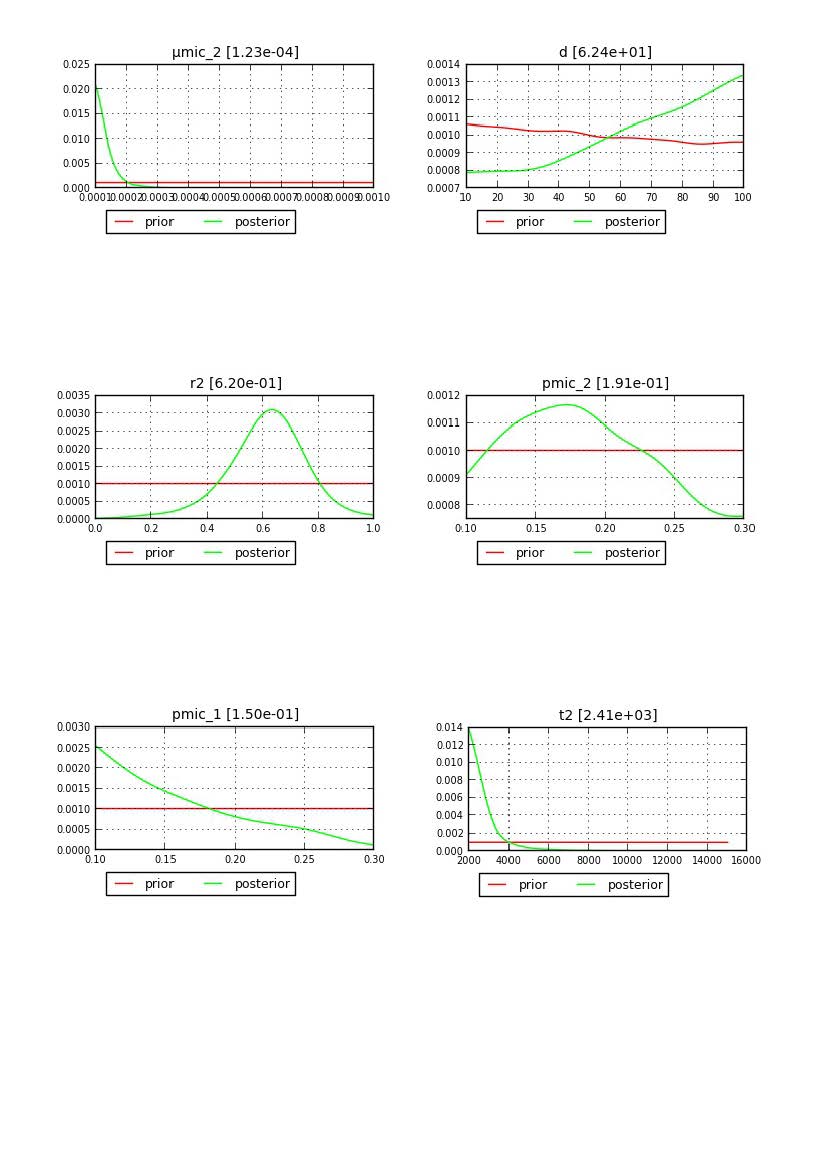


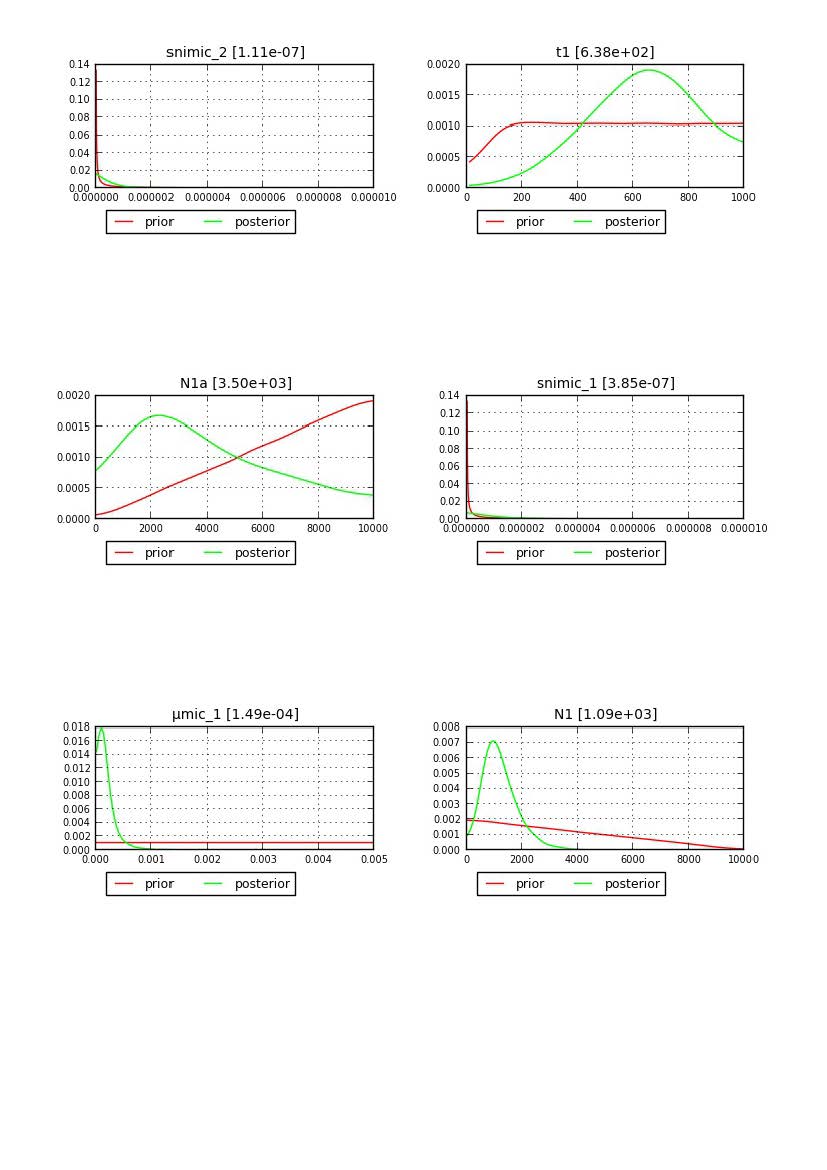


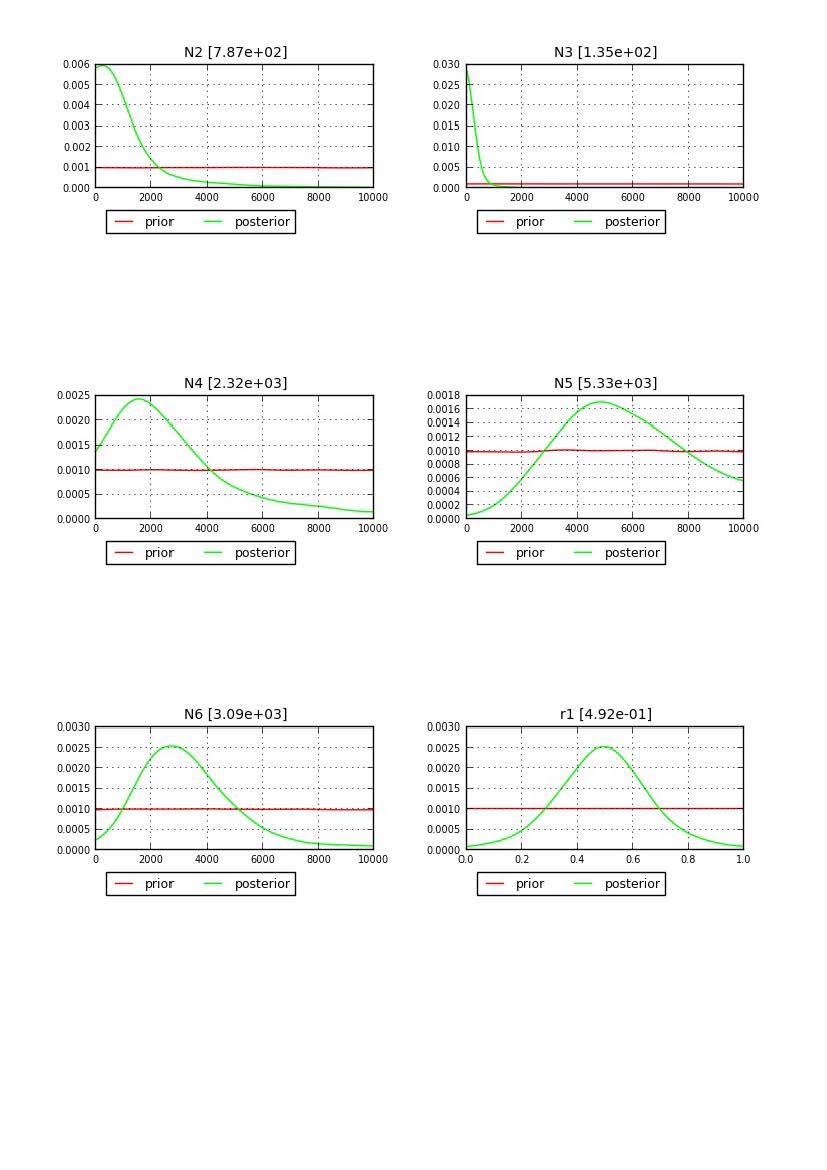


Figure S5. Prior and posterior distributions of 18 parameters incorporated in Scenario 7, the most probable evolutionary model, estimated from DIYABC analysis (See Table1 for population).

Appendix S1.

**The final nine evolutionary scenarios competed in DIYABC**

Scenario 1 (*simple split model*): This is a simple divergence scenario at t2, which assumes divergence of all six groups from the ancestral Chinese population with larger effective population size (Na) than the effective population sizes of all six groups. The divergence event was hypothesized to occur around the LGM, where the major changes in distribution of vegetation throughout east Asia^35^_._

Scenario 2 (*split model with bottlenecks*): The scenario assumes simple divergence at t2 from the ancestral Chinese population with effective population size Na followed by population bottlenecks for all six groups between t2 and t1.

Scenario 3 (*hierarchical split model 1*): Because China is a diversity hotspot of A. cochinchinensis, we believed that the ancestral population likely be placed in China. In the model, the island Taiwanese TPE (pop 6; see Table 1 for the population information) population split at t2 from the Chinese GND (pop1) population derived from the ancestral population (Na) at t2+d. As NAH & YGW (pop5) and OKN (pop3) were genetically more distant from GND, those populations were assumed to coalesce long ago at t2-d and t1+d to the Chinese GND population. The group sharing the most genetic affinities with the GND was just recently diverged from it at t1-d.

Scenario 4 (*hierarchical split model 2*): The model assumes hierarchical split just as model 3 except the split of pop 5. However, in the model 4, considering the genetic affinity of pop 5 to the pop 6 (Taiwanese group, TPE), the pop 5 split from the pop 6 within the recent past at t1-d.

Scenario 5 (*hierarchical split model 3*): In the model, the most genetically close pop 5 split from pop 6 at t1-d. Also, the groups (pops 3 & 4) sharing genetic affinities with both the Chinese GND (pop1) and the Taiwanese TPE (pop6) coalesce to TPE instead of GND sequentially at t1+d and t1.

Scenario 6 (*simple split model associated with unsampled ghost population*): The model assumes split of pop 5 from pop 6 and split of pop 2 from pop 1 within the recent past at t1 based on the genetic affinities. One specific condition of the model is that the model employs a ghost population that is unsampled for the study. To account for scarcity of Chinese (diversity hotspot of A. cochinchinensis) samples, we created a new population that might have contributed to the evolutionary paths of Korean A. cochinchinensis. The model assumes that the groups (pops 3 & 4) sharing genetic affinities with both the Chinese GND (pop1) and the Taiwanese TPE (pop6) might have diversified from the third genetic source (unsampled ghost population, pop7) at t1.

Scenario 7 (*split with admixture model 1*): pops 3 & 4 was set to be admixed by pop1 and pop 6 at t1+d and t1 respectively since the two groups showed pattern of admixture between the two groups, the Chinese GND and the Taiwanese TPE. The remaining groups, pop2 and pop5 were set to split from the pop 1 and pop 6 at t1 just as the other simple split models.

Scenario 8 (*split with admixture model 2*): The model assumes only pop 3 was admixed with pop1 and pop1 at t1+d. The other pop sharing genetic affinities with both pop1 and pop 6, pop 4 was set to be diversified from the admixed pop 3 at t1.

Scenario 9 (*split with admixture model with bottlenecks*): Along with the admixture as in the model 7, the model 9 suggests recent population bottlenecks after split and admixture events at t1-d.
